# Supplementary material for: Mapping the evolution of fertility support policies in China: A content and instrumental analysis
Source: PLoS One. 2025 Oct 9;20(10):e0332137. doi: 10.1371/journal.pone.0332137 (PMC12510515; doi:10.1371/journal.pone.0332137)
Supplement: S1 Appendix — (ZIP) [file pone.0332137.s001.zip › S1 Appendix. 226 original policy documents/217-第九届全国人民代表大会第四次会议关于国民经济和社会发展第十个五年计划纲要及关于纲要报告的决议(FBM-CLI-1-176879).doc]

第九届全国人民代表大会第四次会议关于国民经济和社会发展第十个五年计划纲要及关于纲要报告的决议

**发布部门：** [全国人民代表大会](https://www.pkulaw.com/law/chl?Aggs.IssueDepartment=101)

**发布日期：**2001.03.15

**实施日期：**2001.03.15

**时效性：** [现行有效](https://www.pkulaw.com/law/chl?Aggs.TimelinessDic=01)

**效力级别：** [工作文件](https://www.pkulaw.com/law/chl?Aggs.EffectivenessDic=XA0105)

**法规类别：** [人大议事](https://www.pkulaw.com/law/chl?Aggs.Category=00106)

第九届全国人民代表大会第四次会议关于国民经济和社会发展第十个五年计划纲要及关于纲要报告的决议
（2001年3月15日第九届全国人民代表大会第四次会议通过）

　　第九届全国人民代表大会第四次会议，经过认真审查和审议，决定批准《[中华人民共和国国民经济和社会发展第十个五年计划纲要](https://www.pkulaw.com/chl/f439200c548488c1bdfb.html)》和朱镕基总理代表国务院所作的《关于国民经济和社会发展第十个五年计划纲要的报告》。会议认为，《纲要》和《报告》对“九五”时期的工作总结是实事求是的，提出的今后五年经济和社会发展的奋斗目标、指导方针和主要任务，符合全国各族人民的根本利益，反映了时代发展和建设现代化事业的要求，经过努力是能够实现的。
　　会议认为，过去的五年，全国各族人民在中国共产党领导下，团结奋斗，克服重重困难，取得了巨大成就。第九个五年计划的胜利完成、现代化建设第二步战略目标的提前实现，为实施第十个五年计划、向第三步战略目标迈进，奠定了良好基础。同时也要高度重视经济和社会生活中存在的问题，采取有效措施加以解决。
　　会议指出，实现《纲要》提出的奋斗目标，必须坚持以邓小平理论和党的基本路线为指导，按照“三个代表”重要思想的要求，处理好改革、发展、稳定的关系，坚持发展是硬道理的战略思想，以发展为主题，以结构调整为主线，以改革开放和科技进步为动力，把提高人民生活水平作为根本出发点，推动经济和社会协调发展。
　　会议强调，必须把加强农业基础地位、增加农民收入作为经济工作的首要任务，加快农业和农村经济结构调整，着力促进农业产业化经营，大力推进农村各项改革，切实减轻农民负担，继续抓好农村扶贫工作，积极稳妥推进城镇化。要加大经济结构战略性调整的力度，重点加快工业改组改造和结构优化升级，发展高新技术产业，加强基础设施建设，加快服务业发展。要有步骤、有重点地实施西部大开发战略，加快中西部地区发展。要千方百计扩大就业，不断增加居民收入，深化收入分配制度改革，加快完善社会保障制度，改善城乡居民生活环境，保障人民安居乐业。
　　会议认为，落实科教兴国战略是完成“十五”计划各项任务的重要保证。要促进科技进步和创新，加快科技体制改革。教育要适度超前发展，着力推进素质教育，巩固基本普及九年义务教育的成果，深化教育体制改革。要继续实施可持续发展战略，坚持计划生育的基本国策，保护和合理利用资源，加强生态建设和环境保护，综合治理环境污染，促进人口、资源、环境协调发展。
　　会议强调，必须坚定不移地深化改革，扩大开放。要大力推进体制创新，逐步完善社会主义市场经济体制。继续深化国有企业改革，进一步实行政企分开，切实转变政府职能。坚持公有制经济为主体，支持、鼓励和引导非公有制经济健康发展。继续深化财税、金融、投资体制改革。大力整顿和规范市场经济秩序。不断提高对外开放水平，抓紧做好加入世界贸易组织的各项工作，进一步发展对外贸易，合理有效利用外资。
　　会议要求，积极推进社会主义民主法制建设，发展社会主义民主政治，依法治国，建设社会主义法治国家。继续推进政治体制改革。大力加强社会主义精神文明建设，建立适应社会主义市场经济发展的思想道德体系，把依法治国和以德治国结合起来。坚持和完善民族区域自治制度，发展平等、团结、互助的社会主义民族关系，促进各民族共同繁荣进步。各级人民政府都要依法行政，从严治政，全心全意为人民服务。要深入开展反腐败斗争，健全依法行使权力的监督制约机制。加强社会治安综合治理，依法惩治各种危害社会和国家安全的犯罪活动，依法取缔“法轮功”等邪教组织，进一步维护社会稳定。加强军队建设，增强国防实力。
　　会议指出，要继续按照“一国两制”方针和基本法，维护香港、澳门的长期繁荣稳定。要坚持“和平统一、一国两制”的基本方针和江泽民主席的八项主张，与广大台湾同胞一道，坚决反对和阻止任何制造分裂的图谋，继续推动两岸对话与谈判，发展两岸经济文化交流和人员往来，早日完成祖国统一大业。
　　会议指出，要始终不渝地奉行独立自主的和平外交政策，在和平共处五项原则的基础上，进一步同世界各国发展友好合作关系，推动建立和平稳定、公正合理的国际政治经济新秩序。
　　会议号召：全国各族人民紧密团结在以江泽民同志为核心的党中央周围，高举邓小平理论的伟大旗帜，坚持党的基本路线，以“三个代表”重要思想为指导，万众一心，开拓进取，扎实工作，为实现第十个五年计划，为把我国建设成为富强民主文明的社会主义现代化国家而努力奋斗!
　　附件一：

关于[国民经济和社会发展第十个五年计划纲要](https://www.pkulaw.com/chl/f439200c548488c1bdfb.html)的报告（2001年）
--2001年3月5日在第九届全国人民代表大会第四次会议上
国务院总理　朱镕基

各位代表：
　　从今年开始，我国将实施新世纪第一个五年计划。中国共产党十五届五中全会通过的《[中共中央关于制定国民经济和社会发展第十个五年计划的建议](https://www.pkulaw.com/chl/37b66332069ffbc7bdfb.html)》，提出了未来五年国民经济和社会发展的奋斗目标、指导方针和主要任务。根据《建议》的精神，国务院认真听取各方面意见，制定了《[中华人民共和国国民经济和社会发展第十个五年计划纲要（草案）](https://www.pkulaw.com/chl/f439200c548488c1bdfb.html)》。现在，我代表国务院向大会作报告，请各位代表连同《纲要（草案）》一并审议，并请全国政协各位委员提出意见。
　　**一、**“九五”时期国民经济和社会发展的回顾
　　过去五年，全国各族人民在中国共产党领导下，团结奋斗，开拓创新，各个方面取得了重大成就。
　　国民经济持续快速健康发展，综合国力进一步增强。国内生产总值2000年达89404亿元，平均每年增长8.3%。人均国民生产总值比1980年翻两番的任务，已经超额完成。在经济持续增长和效益改善的基础上，2000年国家财政收入达13380亿元，平均每年增长16.5%。主要工农业产品产量位居世界前列，商品短缺状况基本结束。产业结构调整取得积极进展。粮食等主要农产品生产能力明显提高，实现了农产品供给由长期短缺到总量基本平衡、丰年有余的历史性转变。淘汰落后和压缩过剩工业生产能力取得成效，重点企业技术改造不断推进。信息产业等高新技术产业迅速成长。基础设施建设成绩显著，能源、交通、通信和原材料的“瓶颈”制约得到缓解。
　　经济体制改革全面推进，社会主义市场经济体制初步建立。国有大中型企业建立现代企业制度的改革取得重要进展。大多数国家重点企业进行了公司制改革，其中相当一部分在境内外上市。企业扭亏增盈成效显著，2000年国有及国有控股工业企业实现利润2392亿元，为1997年的2.9倍。国有大中型企业改革和脱困的三年目标基本实现。在公有制经济进一步发展的同时，私营、个体经济有了较快发展。市场体系建设继续推进，资本、技术和劳动力等要素市场迅速发展，市场在资源配置中的基础性作用明显增强。财税体制继续完善。金融改革步伐加快。城镇住房制度、社会保障制度和政府机构等方面改革取得重大进展。国家宏观调控体系进一步健全。
　　对外开放水平不断提高，全方位对外开放格局基本形成。对外经贸体制改革稳步推进，外向型经济迅速发展。2000年进出口总额达4743亿美元，其中出口2492亿美元，分别比1995年增长69%和67%。出口商品结构改善，机电产品和高技术产品所占比重提高。对外开放领域逐步扩大，投资环境继续改善。吸收外资规模增大、质量提高。五年累计实际利用外资2894亿美元，比“八五”时期增长79.6%。国家外汇储备2000年底达1656亿美元，比1995年底增加920亿美元。
　　人民生活继续改善，总体上达到小康水平。农村居民人均纯收入和城镇居民人均可支配收入，2000年分别达到2253元和6280元，平均每年实际增长4.7%和5.7%。市场商品丰富，居民消费水平不断提高，社会消费品零售总额平均每年增长10.6%。城乡居民住房、电信和用电等生活条件有较大改善。居民储蓄存款余额五年增长1倍多，股票、债券等其他金融资产迅速增加。农村贫困人口大幅度减少，“八七”扶贫攻坚目标基本实现。
　　科技、教育加快发展，社会事业全面进步。“863”计划顺利实施。航空航天、信息、新材料和生物工程等高技术领域获得一批重要成果。基础研究和应用研究取得新进展。部门所属应用型科研院所企业化改革基本完成，其他科研院所体制改革全面展开。科技成果市场化、产业化进程加快。各级各类教育全面发展。基本普及九年义务教育和基本扫除青壮年文盲的目标初步实现。高等教育管理体制改革取得重大进展。扩大高校招生受到群众普遍欢迎。人口和计划生育工作取得新成绩。生态建设和环境保护的力度明显加大。文化、卫生、体育等各项社会事业继续发展。廉政建设和反腐败斗争不断取得成效。社会治安综合治理进一步加强。社会主义精神文明建设和民主法制建设取得新的进展。国防和军队建设迈出新的步伐。
　　“九五”期间，我国政府恢复对香港、澳门行使主权，祖国和平统一大业取得历史性进展。香港、澳门回归祖国以来，“一国两制”方针和基本法得到全面贯彻执行。特别行政区政府工作卓有成效，香港、澳门社会稳定，经济发展。
　　随着“九五”计划的完成，我们实现了现代化建设第二步战略目标，为实施“十五”计划、开始迈向第三步战略目标奠定了良好基础。这是我国社会主义现代化事业取得的伟大成就，是中华民族发展史上一个新的里程碑。
　　“九五”时期经济和社会发展的巨大成就，是克服重重困难取得的，来之不易。我们成功应对了国际突发事件的挑战，有效抵御了亚洲金融危机的冲击，既克服“九五”前期通货膨胀的影响，又抑制了中后期的通货紧缩趋势，并战胜了严重的水旱灾害。这些成就的取得，是以江泽民同志为核心的党中央面对诸多矛盾和困难交织的局面，运筹帷幄，及时作出一系列正确决策和部署，全国上下团结奋斗的结果。我代表国务院，向在各个领域和岗位上辛勤劳动、作出贡献的全国各族人民致以崇高敬意!向关心与支持祖国建设和统一的香港特别行政区、澳门特别行政区同胞和台湾同胞以及海外侨胞，表示诚挚的感谢!
　　“九五”时期的实践，丰富了我们按照发展社会主义市场经济的要求，加强和改善宏观经济管理与调控的经验。
　　第一，坚持用发展的办法解决前进中的问题。发展是硬道理。面对各种社会矛盾，我们始终抓住经济建设这个中心，采取有效措施促进国民经济持续快速健康发展，从而为处理好其他矛盾提供了基础。同时，坚持两手抓、两手都要硬的方针，不断加强社会主义精神文明建设和民主法制建设，为集中精力从事经济建设创造了良好的政治社会环境，并提供强大的精神动力。
　　第二，根据经济形势的变化，适时调整宏观调控政策取向和力度。在治理通货膨胀时，注意保持经济持续增长，成功实现“软着陆”。在抑制通货紧缩趋势时，坚持扩大内需的方针，果断实施积极的财政政策，并在实践中不断完善各项政策措施。既增发国债、扩大投资，又适当增加城镇中低收入居民的收入，鼓励增加消费，从两个方面拉动经济增长。采取提高出口退税率和严厉打击走私等措施，千方百计扩大出口，以平衡国际收支，稳定人民币币值。实行稳健的货币政策，运用利率等多种手段支持经济增长，同时注意调节货币供应量，引导信贷投向，防范和化解金融风险。
　　第三，把扩大内需和调整经济结构紧密结合起来。根据一般加工工业生产能力普遍过剩的现实状况，把国债投资重点放在基础设施建设方面，同时增加对农业和科技、教育的投入，支持企业技术改造。利用生产资料生产能力相对富余的时机，办成了一些多年想办而没有办成的大事，既拉动当前经济增长，又增强经济发展后劲。
　　第四，正确处理改革、发展、稳定的关系。在复杂、困难的情况下，改革没有停滞，而是迎难而上，积极有序地向前推进，有力推动了经济发展。同时，始终注意使改革的力度同社会承受能力相适应。在结构调整和改革深化不可避免地触及深层利益关系时，高度重视和采取各种政策措施，维护广大群众的基本利益。大力实施再就业工程，确保国有企业下岗职工基本生活费和离退休人员基本养老金按时足额发放，坚持按保护价敞开收购农民余粮，从总体上维护了社会稳定和经济持续增长。
　　在充分肯定成绩的同时，也要清醒地看到，经济和社会生活中还存在不少问题。主要是：产业结构不合理，地区经济发展不协调；国民经济整体素质不高，国际竞争力不强；社会主义市场经济体制尚不完善，阻碍生产力发展的体制因素仍很突出；科技、教育比较落后，科技创新能力较弱；水、石油等重要资源短缺，部分地区生态环境恶化；就业压力加大，农民和城镇部分居民收入增长缓慢，收入差距拉大；一些领域市场经济秩序相当混乱，重大安全事故时有发生；贪污腐败、奢侈浪费现象和形式主义、官僚主义作风还比较严重；一些地方社会治安状况不好。这些问题产生的原因比较复杂，不少也同我们工作中的缺点和错误有关。我们必须高度重视，进一步采取措施，努力加以解决。

　　**二、**“十五”期间的奋斗目标和指导方针
　　展望新世纪初的国内外形势，未来五到十年，是我国经济和社会发展极为重要的时期。世界新科技革命迅猛发展，经济全球化趋势增强，许多国家积极推进产业结构调整，周边国家正在加快发展。国际环境既对我们提出了严峻挑战，也为我们提供了迎头赶上、实现跨越式发展的历史性机遇。从国内看，我们正处在经济结构调整的关键时期，改革处于攻坚阶段，加入世贸组织又会带来一些新的问题。各方面任务十分繁重，许多深层次矛盾需要解决，形势要求我们必须抓住机遇，加快发展。同时，我们也具备许多有利条件，能够在一个较长时期实现国民经济较快发展。
　　根据“十五”期间的形势和任务，《纲要》提出今后五年经济和社会发展的主要目标是：国民经济保持较快发展速度，经济结构战略性调整取得明显成效，经济增长质量和效益显著提高，为到2010年国内生产总值比2000年翻一番奠定坚实基础；国有企业建立现代企业制度取得重大进展，社会保障制度比较健全，社会主义市场经济体制逐步完善，对外开放和国际合作进一步开展；就业渠道拓宽，城乡居民收入持续增加，物质文化生活有较大改善，生态建设和环境保护得到加强；科技、教育加快发展，国民素质进一步提高，精神文明建设和民主法制建设取得明显进展。
　　“十五”计划《纲要》，体现了以下重要指导方针。
　　坚持把发展作为主题。强调速度与效益相统一，在提高效益的前提下实现较快的发展。有市场、有效益的速度，才是真正的发展，才是硬道理。综合考虑各方面因素，“十五”期间年均经济增长速度预期目标为7%左右。这个速度虽然比“九五”实际达到的速度低一点，但仍然是一个较高的速度。要在提高效益的基础上实现这个目标，必须付出艰巨努力。同时，由于国际国内都存在一些不确定因素，计划的预期目标要留有余地。这样，有利于引导各方面把主要精力放在调整结构和提高效益上，也有利于防止经济过热和重复建设。
　　坚持把结构调整作为主线。我国经济已经到了不调整就不能发展的时候。按原有结构和粗放增长方式发展经济，不仅产品没有市场，资源、环境也难以承受。必须在发展中调整结构，在结构调整中保持较快发展。今后五年要着力调整产业结构、地区结构和城乡结构，特别要把产业结构调整作为关键。要巩固和加强农业基础地位，加快工业改组改造和结构优化升级，大力发展服务业，加快国民经济和社会信息化，继续加强基础设施建设。
　　坚持把改革开放和科技进步作为动力。经济发展和结构调整，都要靠体制创新和科技创新来推动。今后五年要坚定不移地推进改革，扩大开放，突破影响生产力发展的体制性障碍，为经济社会发展提供强大动力。要把发展科技、教育放在突出位置，进一步实施科教兴国战略，振兴科技，培养人才，促进科技、教育与经济紧密结合。
　　坚持把提高人民生活水平作为根本出发点。不断改善城乡人民生活，既是我们发展经济的根本目的，也是扩大内需、促进经济持续增长的迫切需要。要坚持把提高人民生活水平摆在重要位置，扩大就业门路，增加居民收入，合理调节收入分配关系，健全社会保障体系，保证人民群众向更加宽裕的小康生活迈进。
　　坚持把经济发展和社会发展结合起来。大力加强社会主义精神文明建设和民主法制建设，处理好改革、发展、稳定的关系，促进各项社会事业发展，确保社会稳定。高度重视和认真解决人口、资源和生态环境问题，进一步实施可持续发展战略，推动经济、社会、生态环境协调发展。
　　“十五”计划《纲要》突出了战略性、宏观性、政策性，减少实物指标，增加反映结构变化的预期指标；围绕要解决的主要问题和重点发展领域，提出努力方向和相应的政策措施。强调计划的实施要充分发挥市场机制的作用，政府宏观调控要更多地运用经济杠杆、经济政策和法律手段。在计划制定方法上，力求提高社会参与度，使计划制定过程成为发扬民主、集思广益的过程，成为各有关方面达成共识的过程。

　　**三、**加强农业基础地位，努力增加农民收入
　　农业、农村和农民问题是关系改革开放和现代化建设全局的重大问题。“十五”期间要把全面贯彻党在农村的基本政策，加强农业基础地位和增加农民收入，作为经济工作的首要任务，着重抓好以下几项工作。
　　加快农业和农村经济结构调整。这是提高农业经济效益，增加农民收入的根本途径。在切实保护耕地，稳定粮食生产能力的同时，以优化品种、提高质量、增加效益为中心，积极调整种植业结构，加快发展畜牧业、林业、水产业。发挥各地农业的比较优势，合理调整农业生产的区域布局，发展特色农业，形成规模化、专业化的生产格局，提高商品率。大力促进农业产业化经营，扶持龙头企业，推广“公司加农户”、“订单农业”等多种形式，发展农产品加工、储运、保鲜等产业，提高农业的后续效益。引导乡镇企业结构调整、技术进步和体制创新。发展小城镇，繁荣小城镇经济，积极稳妥地推进城镇化，拓宽农民的就业空间和增收渠道。大力推进科教兴农，加强生物技术、信息技术等高新技术的研究与开发应用，推广优良品种和先进适用技术，积极扶持农业科技产业发展。加强农业社会化服务体系建设。建立农产品市场信息、食品安全和质量标准与检测体系。调整结构要因地制宜，依据市场规律，维护农民生产经营自主权，不搞强迫命令。
　　积极推进农村各项改革。在长期稳定土地承包关系的基础上，鼓励有条件的地区积极探索土地经营权流转制度改革。根据粮食生产和流通的新情况，继续深化粮食流通体制改革。在适当扩大中央储备粮规模的同时，粮食主销区要加快粮食流通市场化进程，通过与粮食主产区建立长期稳定的购销关系，以及中央储备粮的轮换与调控，保证粮食需要。粮食主产区要继续坚持“三项政策、一项改革”，按保护价敞开收购农民余粮，中央财政继续给予资金支持，以充分发挥其粮食生产的优势。这样做，既可为主产区腾出粮食销售市场，促进粮价合理回升，也有利于推进主销区农业结构调整，全面增加农民收入。要保护好基本农田，决不允许擅自将耕地改为非农用地，这是一条不可逾越的“红线”。推进农村税费制度改革，取消乡统筹、村提留和其他面向农民征收的一切行政性收费，同时适当提高现行农业税和农业特产税税率。这是保障农民合法权益，减轻农民负担的治本之策。要在总结安徽省试点经验的基础上，加快改革步伐。同税费改革相结合，精简乡镇机构，裁减人员，减少村组干部补贴人数；有条件的地方，适度撤并乡镇。继续深化农村金融改革，积极探索适应农村经济发展要求的农村金融体系。因地制宜加快农村信用社管理体制改革，关键是要明确产权关系，完善法人治理结构，落实防范和化解风险的责任。农村信用社要坚持为农业、农村、农民服务的方向，加强经营管理，充分发挥在农村金融中的主力军和联系农民的金融纽带作用。农业银行和其他金融机构要加强对农业和农村经济的支持。深化农村供销社改革。
　　加强农业和农村基础设施建设。进一步增加投入，加快大江大河大湖治理，抓紧主要江河控制性工程建设和病险水库加固，提高防洪调蓄能力。着力抓好大型灌区节水工程改造，积极开展群众性农田水利建设，搞好水土保持。加强国家商品粮和优质农产品基地建设，搞好农业综合开发。继续加强农村电网、通信、广播电视、道路、供水等设施建设，切实改善农村生产、生活和市场条件。
　　继续搞好农村扶贫工作。“八七”扶贫攻坚任务虽已基本完成，但从根本上改变贫困地区面貌，仍是一项长期而艰巨的任务。要继续加大扶贫力度，重点做好中西部少数民族地区、革命老区、边疆地区和特困地区的扶贫工作。坚持开发式扶贫，多方面增加扶贫资金投入，扩大以工代赈规模，支持贫困地区加强基础设施建设。

　　**四、**大力推进产业结构优化升级
　　产业结构调整和升级，是经济结构战略性调整的重点，要突出以下几个方面。
　　用高新技术和先进适用技术改造提升传统产业。要把传统产业的改组改造放在重要位置，以市场为导向，以企业为主体，以技术进步为支撑，抓好几个环节。一是围绕增加品种、改善质量、节能降耗、防治污染和提高劳动生产率，在能源、冶金、化工、轻纺、机械、汽车、建材及建筑等行业，支持一批重点企业技术改造，切实提高工艺和装备水平。二是通过自主创新和引进技术，加快对推动结构升级的共性技术、关键技术和配套技术的开发。振兴装备制造业，开发制造急需的大型高效和先进成套技术装备。三是通过上市、兼并、联合、重组等形式，在主要行业形成若干拥有自主知识产权、主业突出、核心能力强的大公司和企业集团，使之成为调整结构、促进升级的骨干和依托。四是积极支持和促进老工业基地改造，充分发挥其基础雄厚、人才聚集的优势，努力提高产业水平。以资源开采为主的城市和大矿区，要因地制宜发展接续和替代产业。
　　与此同时，要综合运用经济、法律和必要的行政手段，继续依法关闭产品质量低劣、浪费资源、污染严重、不具备安全生产条件的厂矿，淘汰落后和压缩过剩生产能力，并严禁转移重建。长期亏损、资不抵债、扭亏无望的企业和资源枯竭的矿山，要实施破产关闭。积极疏通和逐步规范企业退出市场的通道。
　　发展高新技术产业，以信息化带动工业化。从实际出发，有选择地加快信息技术、生物工程和新材料等高新技术产业发展。重点支持建设高速宽带信息网络、关键集成电路、新型运载火箭等重大高技术工程，形成我国高新技术产业的群体优势和局部强势。加快发展信息产品制造业，提高自主开发能力和系统集成能力。积极发展软件产业。加强信息基础设施建设。要在企业技术开发和生产营销、社会公共服务、政府行政管理等方面广泛应用数字化、网络化技术，把工业化和信息化更好地结合起来。
　　加强水利、交通、能源等基础设施建设，高度重视资源战略问题。水资源短缺，是我国经济和社会发展的严重制约因素。要把节水放在突出位置，建立合理的水资源管理体制和水价形成机制，全面推行各种节水技术和措施，发展节水型产业，建立节水型社会。加强水污染防治，搞好污水处理和利用。加紧南水北调等重大项目的规划和建设。要加强公路、铁路、港口、航道、机场、管道系统的建设，建立健全畅通、安全、便捷的现代综合运输体系。能源特别是石油问题，是资源战略的一个重要问题。国内石油开发和生产不能适应经济和社会发展的需要，供需矛盾日益突出。必须千方百计节约和替代石油，加快石油和天然气勘探与开发，积极利用国外资源，并尽快建立石油等战略资源的储备制度。积极推进大型煤矿改造，建设高产高效矿井，重视洁净煤技术的开发利用。充分利用现有发电能力，积极发展水电、坑口大机组火电，压缩小火电，适度发展核电。加强城乡电网建设和改造，推进全国联网。深化电力体制改革，逐步推进厂网分开、竞价上网。重视发展各种新能源。
　　加快发展服务业。这是促进结构升级和增加就业的重要途径。积极发展信息、金融、会计、咨询、法律服务等现代服务业，提高服务业整体水平。运用现代经营方式和服务技术，改造商贸流通、交通运输、市政服务等传统服务业，提高服务质量和效益。发展面向居民消费的房地产业、社区服务、旅游、餐饮、娱乐、健身等产业，增加服务内容。要深化改革和采取必要的政策措施，形成有利于服务业发展的环境。

　　**五、**实施西部大开发，促进地区协调发展
　　实施西部大开发战略，加快中西部地区发展，是我国迈向现代化建设第三步战略目标的重要部署。“十五”期间要突出重点，搞好开局，着重加强基础设施和生态环境建设，力争五到十年内取得突破性进展，同时使科技、教育有较大发展。
　　要集中力量建设西气东输、西电东送、青藏铁路等一批具有战略意义的重大项目。把水资源的保护、节约和开发放在突出位置，加强规划，合理配置，努力提高水的利用效率。有步骤而因地制宜推进天然林保护、退耕还林还草以及防沙治沙、草原保护等重要工程的建设，注意发挥生态的自我修复能力，逐步建成我国西部牢固的绿色生态屏障。积极发展教育事业，加快培养急需的各类人才。增加科技投入，提高科技开发能力。要从各地实际出发，调整和优化产业结构，加强农业，加快资源优势向经济优势转化，培育和形成各具特色的地区经济。西部开发要依托亚欧大陆桥、长江水道、西南出海通道等主要交通干线，发挥中心城市的集聚功能和辐射作用，以线串点，以点带面，培育西陇海兰新线经济带、长江上游经济带和南（宁）贵（阳）昆（明）等经济区，带动周围地区发展。
　　国务院已颁布重点支持西部大开发的政策措施，国家将增加对西部地区的投入和财政转移支付。西部地区要立足于主要依靠自己的力量，发扬艰苦创业精神，作好长期奋斗的准备；加快改革开放步伐，营造良好的投资环境，更多更好地吸引境内外资金、技术和人才参与西部开发。要加强干部交流。
　　中部地区要发挥区位优势和综合资源优势，加快经济发展步伐。以主要水陆交通干线地区为重点，发挥中心城市作用，积极培育新的经济增长点和经济带。巩固和发展农业，继续加强基础设施和生态环境建设。加大用高新技术和先进适用技术改造传统产业的力度，提高技术水平和竞争能力。
　　东部沿海地区要面向国内外市场，加快科技进步和创新，着力发展高新技术产业和外向型经济，提高经济的综合素质和国际竞争力，有条件的地区要率先基本实现现代化。要采取多种形式，加强与中部、西部地区的经济技术合作，支持和带动中西部经济发展，拓展东部结构调整和经济增长的空间。

　　**六、**落实科教兴国战略，大力开发人才资源
　　这是《纲要》的重要内容，也是实现今后五年各项任务的重要保证。
　　促进科技进步和创新，为结构调整和经济发展提供强大动力。一要积极推进具有战略意义的高技术研究，力争在一些关系国民经济命脉和国家安全的关键技术领域取得突破。努力提高自主创新能力，促进高新技术成果产业化。二要为传统产业升级提供技术支持。重点在农产品加工及转化、装备制造、节水节能、纺织品后整理等方面的技术取得进展，加快高新技术向传统产业渗透。三要加强基础研究和应用基础研究，提高科技持续创新能力。加强基础科学重点领域的前沿性交叉性研究。选择我国具有优势和对发展有重大意义的领域，加强应用基础研究，力争在基因组学、信息科学、纳米科学、生态科学和地球科学等方面取得新进展。促进自然科学与社会科学的交叉融合，推动管理科学发展。重视发展哲学社会科学，推进理论创新。
　　加快科技体制改革步伐，进一步促进科技与经济紧密结合。加强国家创新体系建设。推动企业成为技术进步和创新的主体。继续鼓励技术开发类科研院所进入企业或改制为企业。积极推进社会公益类科研院所改革。培育和形成一批具有国际影响的科研机构。发展社会化的科技中介服务业。完善风险投资机制，建立创业板股票市场，支持中小企业技术创新。加大国家和社会对科技的投入。加强国家重点实验室建设。
　　坚持教育适度超前发展，为国民经济和社会发展服务。发展教育，要面向现代化、面向世界、面向未来，着力推进素质教育，促进学生德、智、体、美全面发展。巩固基本普及九年义务教育和基本扫除青壮年文盲的成果，加快高中阶段教育和高等教育的发展，重点建设一批高水平大学和学科。大力发展职业教育和职业培训，建立职业教育与普通教育相互沟通的教育体系。发展成人教育和多种形式的继续教育，逐步形成终身教育体系。重视发展儿童早期教育。利用信息技术发展远程教育。根据经济和社会发展的要求，继续调整教育结构和布局，优化专业设置，更新教材，改革课程体系、考试评价制度和教学方法，提高教学质量。加强德育，尤其要重视青少年的品德教育，改进学校思想政治工作。搞好教师队伍建设，全面提高教师的思想和业务素质。
　　深化办学体制和教育管理体制改革。依法落实高校办学自主权，继续推进高校后勤服务社会化。鼓励、支持和规范社会力量办学。增加国家和社会对教育的投入。适应农村税费改革后的新情况，县级政府要负责对基础教育经费的统筹，落实教师工资统一发放的措施。中央财政和省级财政要加大对困难地区、民族地区教育的转移支付和专项投入。健全奖学金、助学金和助学贷款等制度。采取有力措施，切实制止学校乱收费。
　　实施人才战略，把培养、吸引和用好人才作为一项重大任务。着眼于现代化建设全局和长远发展，培养和造就坚持走有中国特色社会主义道路，有较高政治理论素养和开拓精神，掌握现代科学文化和管理知识，并经过实践考验的高素质领导人才队伍。培养和造就具有公仆意识，廉洁、勤政、高素质、专业化的公务员队伍。培养和造就掌握先进科学技术和管理知识、创新能力强、适应经济和社会发展需要的各类专业人才队伍和企业经营管理者队伍。重视培养具有世界前沿水平的学科带头人。普遍提高全体劳动者的科学素养和劳动技能。深化干部人事制度改革，建立和完善对各级各类人才的选拔任用、考核评价、流动配置、激励监督的制度，形成有利于人才辈出、人尽其才的机制。建立和完善人才市场。依法保护知识产权。吸引聘用海外高级专门人才。鼓励留学人员回国工作或以适当方式为祖国服务。

　　**七、**进一步深化改革和扩大对外开放
　　要按照逐步完善社会主义市场经济体制，促进结构调整和经济发展的要求，积极推进改革，扩大对外开放。
　　继续深化国有企业改革，使企业真正成为市场竞争的主体。重点是加快建立和完善现代企业制度。鼓励国有大中型企业通过规范上市、中外合资和相互参股等形式，实行股份制，转换经营机制。对关系国民经济命脉和国家安全的重要企业，国家必须控股；对其他企业，不必都要控股。积极探索国有资产管理的有效形式。健全企业法人治理结构。特别要建立和完善对国有企业的监管机制，充分发挥监事会的作用。深化企业人事、劳动和分配制度改革，建立健全激励机制和约束机制，强化科学管理。要结合产业结构调整，坚持“有进有退、有所为有所不为”的方针，推进国有经济布局的战略性调整。继续放开搞活国有中小企业。进一步实行政企分开，切实转变政府职能，减少行政性审批。发挥商会、行业协会等中介组织的作用。推进电力、铁路、民航、通信等行业管理体制改革，引入竞争机制。通过改革，使政府与企业的关系真正转到符合社会主义市场经济要求的轨道上来。继续调整和完善所有制结构。坚持以公有制经济为主体，发挥国有经济主导作用，发展多种形式的集体经济，支持、鼓励和引导私营、个体经济健康发展。
　　大力整顿和规范市场经济秩序，进一步发展市场体系。这既是保证当前经济正常运行的迫切需要，又是完善社会主义市场经济体制的重要举措。要健全市场法律法规，严格执法。完善市场监督机制，运用现代科技手段，加大监管力度。坚持不懈地打击制售假冒伪劣产品、偷税、骗税、骗汇、走私等违法犯罪活动。整顿建筑市场。整顿和规范金融秩序。严肃财经纪律。加强审计监督工作。规范社会中介机构的行为。强化安全生产管理和安全监察。打破部门、行业垄断和地区封锁，反对地方保护主义，尽快建立和完善全国统一、公平竞争、规范有序的市场体系。重点培育和发展要素市场，尤其是资本市场。大力倡导诚实守信的职业道德，加快建立健全社会信用制度。
　　加强和改善宏观调控，继续深化财税、金融、投资体制改革。根据经济形势变化，实施相应的宏观经济政策。近期要继续实施积极的财政政策，带动投资和促进消费。全面推进依法理财，强化税收征管，严格财政监督，加快税费改革和预算制度改革，健全税制。调整和优化财政支出结构，逐步建立适应社会主义市场经济要求的公共财政框架。在继续扩大国内需求和抑制通货紧缩趋势的情况下，注意防止可能出现的经济过热和通货膨胀。继续实行稳健的货币政策，适时调节货币供应量，保持人民币币值稳定。按照现代银行制度对国有独资商业银行进行综合改革，发挥政策性银行功能，办好中小金融机构。规范和健全证券市场，保护投资者利益。进一步发展保险业。改善和强化金融监管，实行严格的经营管理考核制度和责任追究制度，改进金融服务，努力提高金融资产质量，防范和化解金融风险。深化投资体制改革，全面实行投资项目法人责任制、招标投标制、工程监理制、合同管理制，健全投资约束机制。
　　适应经济全球化趋势，进一步提高对外开放水平。一要抓紧做好加入世贸组织的准备和过渡期的各项工作。采取切实措施，转变政府管理方式，提高企业竞争能力。深化改革，健全符合国际通行规则和我国国情的对外经济贸易体制。加快修订和完善相关的法律法规。抓紧培养熟悉国际贸易规则的专业人才。二要进一步发展进出口贸易。继续实施以质取胜、科技兴贸战略。优化出口商品结构，提高高新技术产品比重，增加大宗传统商品的技术含量和附加值，扩大服务贸易规模。规范加工贸易管理，提高加工贸易的增值率。大力推进市场多元化战略，开拓新的出口市场。同时，重点进口国内急需的先进技术、关键设备和重要原材料。积极参与多边贸易体系和国际区域经济合作。三要努力提高利用外资水平。有步骤地推进服务领域对外开放。鼓励外商特别是跨国公司投资高新技术产业、基础设施等领域，以及在我国建立研究开发机构，参与国有企业的改组改造。支持有条件的企业到境外上市。进一步改善投资环境。积极探索采用收购、兼并、风险投资、投资基金和证券投资等形式，扩大利用外资规模。积极引导外资更多地投向中西部地区。要继续办好经济特区、浦东新区。四要实施“走出去”战略。鼓励有比较优势的企业到境外投资，开展加工贸易，合作开发资源，发展国际工程承包，扩大劳务出口等。建立和完善政策支持体系，为企业到境外投资兴业创造条件。同时要加强监管，防止国有资产流失。

　　**八、**不断改善人民生活，完善社会保障制度在经济发展的基础上，使人民生活有较大改善，逐步向更加宽裕的小康生活迈进，是今后五年的重要任务。
　　加快完善社会保障制度。这是关系改革、发展、稳定全局的大事。要确保国有企业下岗职工基本生活费和离退休人员基本养老金按时足额发放。同时，加快形成独立于企业事业单位之外、资金来源多元化、保障制度规范化、管理服务社会化的社会保障体系。精心做好完善城镇社会保障体系的试点工作。完善社会统筹和个人帐户相结合的城镇职工基本养老保险制度。健全失业保险制度，逐步实现国有企业下岗职工基本生活保障和失业保险并轨。加强城市居民最低生活保障制度，关心困难群众的生活。积极推进城镇职工基本医疗保险制度、医疗机构和药品流通体制改革。鼓励有条件的用人单位为职工建立补充养老和医疗保险，并发挥商业保险的作用。建立可靠、稳定的社会保障资金筹措、有效运营和严格管理机制。发展社会福利、社会救济、优抚安置和社会互助等社会保障事业。切实保障妇女、未成年人、老年人、残疾人的合法权益。支持残疾人事业发展。
　　千方百计扩大就业。“十五”期间扩大就业的任务十分繁重。在保持较快经济增长的同时，要重视发展有比较优势的劳动密集型产业，特别是发展就业容量大的服务业，积极发展集体企业和私营、个体企业，努力增加就业岗位，拓宽就业渠道。加强社区建设，扩大社区服务。发展灵活多样的就业形式。引导人们转变就业观念，鼓励创业和自谋职业。加强劳动力市场建设，完善就业服务体系，发展职业培训，形成市场导向的就业机制。
　　不断增加居民收入，特别是增加低收入者的收入。建立健全与经济增长相适应的工资支付制度、最低工资制度和最低工资标准调整机制。各级财政要按照“一要吃饭、二要建设”的原则，保证机关事业单位工作人员工资和离退休费按时足额发放，逐步改善他们的工资待遇。深化收入分配制度改革。坚持效率优先、兼顾公平原则。认真贯彻按劳分配为主体、多种分配方式并存的制度，鼓励资本、技术等生产要素参与收益分配。要提高国有企业高层管理人员、技术人员的工资报酬，充分体现他们的劳动价值，可以试行年薪制。但同时要有严格的约束和监督，不能不管工作好坏、企业效益高低，都照拿高薪。对不称职、渎职的人要有相应的制裁措施。对国有上市公司负责人和技术骨干，还可以试行期权制，但不能把国有资产量化给个人。加强对垄断行业个人收入分配的监管。要规范社会分配秩序，强化税收对收入分配的调节功能，防止收入差距过分扩大。
　　调整消费结构，改善消费环境。着力改善城乡居民的住行条件。大力发展经济适用住房，建立廉租房供应保障体系。积极发展公共交通。加强城市绿化和城乡基础设施建设，改善城乡居民生活环境。发展社区卫生预防保健，努力改善农村卫生服务条件。加强群众性体育设施建设。

　　**九、**继续实施可持续发展战略
　　促进人口、资源、环境协调发展，把实施可持续发展战略放在更突出的位置。
　　坚持计划生育基本国策。要努力保持低生育水平，促进优生优育。重点做好农村和流动人口计划生育工作。建立计划生育利益导向机制，继续实行地方党政领导责任制。加快人口与计划生育法制建设。认真做好老龄人口的工作，发展老龄事业。
　　保护和合理利用资源。依法保护和合理利用淡水、土地、能源等宝贵资源。逐步建立战略性矿产资源储备和安全供应体系。加强对海洋资源的综合开发利用和保护。积极开展资源回收利用，大力提高资源综合利用率。健全资源的有偿使用制度。维护矿产等资源的国家所有者权益。完善资源保护和利用的法律法规，强化执法监督。
　　加强生态建设和环境保护。抓好长江上游、黄河上中游等地区的天然林保护工程建设。继续加强东北、华北、西北和长江中下游等重点防护林体系建设。加强天然草原的保护和建设。推进岩溶地区石漠化综合治理。抓紧治理京津地区风沙源。继续开展重点流域、区域、海域的污染治理。综合治理城市污染，搞好城市绿化，使大中城市环境质量明显改善。重视农村污染治理和环境保护。健全环境、气象和地震监测体系，做好防灾减灾工作。

　　**十、**加强精神文明和民主法制建设，加强国防建设
　　大力加强社会主义精神文明建设。坚持两手抓、两手都要硬的方针。巩固和加强马克思主义的指导地位，用马列主义、毛泽东思想、邓小平理论教育人民，弘扬爱国主义、集体主义、社会主义精神。在干部中深入开展“三个代表”重要思想的教育，坚持讲学习、讲政治、讲正气，树立正确的世界观、人生观、价值观。努力建立适应社会主义市场经济发展的思想道德体系，把依法治国和以德治国结合起来。普及科学知识，反对愚昧迷信，倡导文明健康的生活方式。进一步发展文学艺术、新闻出版、广播影视等各项事业。坚持为人民服务、为社会主义服务的方向和百花齐放、百家争鸣的方针，生产出更多更好的精神产品。坚持正确的舆论导向。高度重视新的信息媒体的建设和管理。加强图书馆、文化馆、科技馆、博物馆、档案馆等文化设施建设，加强青少年、老年活动场所建设。继续开展群众性精神文明创建活动。深化文化体制改革，完善文化经济政策，推动有关文化产业发展。整顿和规范文化市场。坚持不懈地开展“扫黄打非”斗争。
　　积极推进社会主义民主法制建设。发展社会主义民主政治，依法治国，建设社会主义法治国家。继续推进政治体制改革。实行民主选举、民主决策、民主管理和民主监督，保证人民依法享有广泛的权利和自由，尊重和保障人权。继续加强人民代表大会的立法和监督工作。发挥人民政协的政治协商、民主监督、参政议政作用。完善适应社会主义市场经济体制的法律体系。各级政府要自觉接受同级人大的监督，主动听取政协的意见。推进政府工作法制化，依法行政，从严治政。深化司法改革，严格执法，公正司法。深入开展反腐败斗争，加强廉政建设。强化法制教育，增强全体公民的法制观念。加强政法系统基础设施建设，提高政法队伍素质。
　　进一步落实党的民族政策，坚持和完善民族区域自治制度，发展平等、团结、互助的社会主义民族关系。结合西部大开发，加大支持力度，加快少数民族和民族地区经济与社会全面发展，促进各民族共同繁荣和进步。全面贯彻党的宗教政策，保障公民宗教信仰自由，依法管理宗教事务，积极引导宗教与社会主义社会相适应。认真贯彻党的侨务政策，充分发挥海外侨胞在祖国现代化建设与和平统一大业中的作用。
　　进一步维护社会稳定。正确处理新时期人民内部矛盾。重视群众的来信来访。搞好社会治安综合治理，打击各种危害社会与国家安全的犯罪活动。依法打击民族分裂活动、宗教极端势力、暴力恐怖活动、邪教和利用宗教进行非法活动。继续开展同“法轮功”邪教组织的斗争，深入揭露和批判其反人类、反社会、反科学的邪教本质，及其沦为国内外敌对势力反对我国社会主义政权工具的反动面目，依法严厉打击极少数违法犯罪分子，坚持不懈地团结、教育、挽救绝大多数被蒙骗的群众。
　　加强国防和军队建设。这是国家安全和现代化建设的重要保证。在集中力量发展国民经济的同时，必须积极推进国防建设，增强国防实力。要大力加强军队思想政治建设，确保党对军队的绝对领导和军队建设的正确方向。认真贯彻新时期积极防御的战略方针，坚持改革创新，坚持质量建军，科技强军，勤俭建军，依法治军，走有中国特色的精兵之路，全面提高军队革命化、现代化、正规化建设水平。强化全民国防意识，健全国防动员体制。加强国防科研，推进国防科技工业调整、改革，发展新型武器装备，增强现代技术特别是高技术条件下防卫作战的能力，做好应付各种复杂情况的准备。
　　各位代表!进入新世纪，我们将继续按照“一国两制”方针和基本法，全力支持香港、澳门特别行政区行政长官和政府依法施政，加强港澳与内地的经济合作及交流，维护香港、澳门的长期繁荣稳定。
　　尽早解决台湾问题，实现祖国完全统一，是中华儿女的共同期盼，是我们面临的重大任务。我们将继续执行“和平统一、一国两制”的基本方针和江泽民主席提出的八项主张，与广大台湾同胞一道，坚决阻止任何制造分裂的图谋，尽一切可能争取和平统一。我们将坚持一个中国原则，在此基础上继续推动两岸对话与谈判，发展两岸经济文化交流和人员往来。我们坚信，经过中华儿女的不懈奋斗，祖国统一大业必将早日完成。
　　面对世界多极化和经济全球化深入发展的国际形势，我们将始终不渝地奉行独立自主的和平外交政策，在和平共处五项原则的基础上，进一步同世界各国发展友好合作关系，争取一个长期的国际和平环境和良好的周边环境。中国将积极参与国际事务，为维护世界和平、促进国际合作、实现共同发展，为推动建立和平稳定、公正合理的国际政治经济新秩序，作出新的贡献。
　　各位代表!2001年是进入新世纪、实施“十五”计划的第一年。全面贯彻党的十五届五中全会和中央经济工作会议精神，做好今年的各项工作，对于实施“十五”计划起好步、开好局，至关重要。要坚持扩大国内需求的方针，实施积极的财政政策和稳健的货币政策。继续发行1500亿元长期建设国债，集中用于在建项目和西部开发项目。同时，适当增加机关事业单位人员工资，提高城镇低收入者收入。把加强农业基础地位和增加农民收入放在经济工作首位，采取有力措施切实解决当前农业、农村中存在的突出问题。巩固和扩大国有企业改革成果，在建立现代企业制度、转换经营机制和加强科学管理上狠下功夫。抓紧做好加入世贸组织的各项准备工作。加快完善社会保障体系，多渠道扩大就业，改善人民生活。要把大力整顿和规范市场经济秩序，作为一项紧迫的重要任务。加强精神文明和民主法制建设。继续处理好改革、发展、稳定的关系，搞好社会治安综合治理，维护社会稳定。支持北京市申办奥运。要统筹兼顾，乘势前进，保持经济和社会发展的良好势头。
　　各位代表!我们已经跨进了21世纪，迈上了向现代化建设第三步战略目标进军的新征程。任何艰难险阻都挡不住中国人民胜利前进的步伐！让我们紧密团结在以江泽民同志为核心的党中央周围，高举邓小平理论伟大旗帜，坚持党的基本路线，以“三个代表”重要思想为指导，振奋精神，扎实工作，为实现“十五”计划目标，为把我国建设成为富强民主文明的社会主义现代化国家而努力奋斗！
　　附件二：

　　《关于国民经济和社会发展第十个五年计划纲要的报告》修改情况说明

九届全国人大四次会议主席团：
　　根据出席九届全国人大四次会议代表审议和全国政协九届四次会议委员讨论时提出的意见和建议，国务院对《关于国民经济和社会发展第十个五年计划纲要的报告》进行了反复推敲和认真修改，共修改40处，其中涉及内容、比较重要的改动有23处。现将主要情况报告如下。
　　1、在《报告》的第三部分关于农业和农村经济结构调整的一段中，增加了“加强农业社会化服务体系建设”的内容；在“发展小城镇”一句之后，增加了“繁荣小城镇经济”。在关于扶贫工作的一段中，增加了“继续加大扶贫力度”一句。
　　2、在第四部分关于改造和提升传统产业的一段中，增加了“以资源开采为主的城市和大矿区，要因地制宜发展接续和替代产业”。在关于基础设施建设和资源战略问题的一段中，将“建立合理的水价形成机制”改为“建立合理的水资源管理体制和水价形成机制”；在“加强水污染防治”之后，加上了“搞好污水处理和利用”；把“千方百计节约石油”改为“千方百计节约和替代石油”；在电力发展方面，加了“加强城乡电网建设和改造，推进全国联网”一句。
　　3、在第五部分关于西部地区发展的一段中，增加了“加快资源优势向经济优势转化”。在关于中部地区发展的一段中，增加了“发挥中心城市的作用”。
　　4、在第六部分关于教育发展的两段中，增加了“重视发展儿童早期教育”；在“加强德育”之后，增加了“尤其要重视青少年的品德教育”；把有关财政加大对教育经费投入的表述改为：“适应农村税费改革后的新情况，县级政府要负责基础教育经费的统筹，落实教师工资统一发放的措施。中央财政和省级财政要加大对困难地区、民族地区教育的转移支付和专项投入。”
　　5、在第七部分关于整顿和规范市场秩序、发展市场体系的一段中，加了“反对地方保护主义”一句；在关于宏观调控和财税、金融、投资体制改革的一段中，加了H“健全税制”的内容。在关于对外开放的一段中，增加了“积极引导外资更多地投向中西部地区。要继续办好经济特区、浦东新区。”
　　6、在第八部分关于千方百计扩大就业的一段中，增加了“加强社区建设，扩大社区服务”。
　　7、在第九部分关于加强生态和环境保护的一段中，增加了“加强天然草原的保护和建设”，“搞好城市绿化”的内容；把“重视农村环境保护，特别要防治农业化学污染”改为“重视农村污染治理和环境保护。”
　　8、在第十部分关于精神文明建设的一段中，增加了“继续开展群众性的精神文明创建活动”，“完善文化经济政策”的内容。
　　在“全力支持香港、澳门特别行政区行政长官和政府依法施政”一句之后，增加了“加强港澳与内地的经济合作及交流”。
　　需要说明的是，讨论中有的代表和委员建议，删去《纲要（草案）》和《报告》中关于“继续加强人民代表大会的立法和监督工作”和“发挥人民政协的政治协商、民主监督、参政议政作用”的内容。我们对此进行了认真研究，认为这些内容应予保留。主要考虑是：一、《纲要（草案）》是整个国家国民经济和社会发展的计划纲要，其内容应涵盖包括国家根本政治制度的完善与发展在内的社会进步的各个方面；二、《纲要（草案）》是根据中共中央关于制定“十五”计划的《建议》制定的，上述有关内容是引自《建议》的表述，“九五”计划《纲要》也有这方面的内容；三、《报告》是对《纲要（草案）》的说明，不同于一般的政府工作报告，所以引述了《纲要（草案）》有关这方面的内容；四、国务院提出《纲要（草案）》及其《报告》，提请全国人民代表大会审议批准后，即成为国家的意志，成为国家正式的国民经济和社会发展第十个五年计划的纲要。

国务院总理　朱镕基
2001年3月10日

　　附件三：

[中华人民共和国国民经济和社会发展第十个五年计划纲要](https://www.pkulaw.com/chl/f439200c548488c1bdfb.html)
（2001年3月15日第九届全国人民代表大会第四次会议批准）

　　目录

　　序言
　　第一篇指导方针和目标
　　第一章　国民经济和社会发展的指导方针
　　第二章　国民经济和社会发展的主要目标
　　第二篇经济结构
　　第三章　加强农业基础地位，促进农村经济全面发展
　　第四章　优化工业结构，增强国际竞争力
　　第五章　发展服务业，提高供给能力和水平
　　第六章　加速发展信息产业，大力推进信息化
　　第七章　加强基础设施建设，改善布局和结构
　　第八章　实施西部大开发战略，促进地区协调发展
　　第九章　实施城镇化战略，促进城乡共同进步
　　第三篇科技、教育和人才
　　第十章　推进科技进步和创新，提高持续发展能力
　　第十一章　加快教育发展，提高全民素质
　　第十二章　实施人才战略，壮大人才队伍
　　第四篇人口、资源和环境
　　第十三章　控制人口增长，提高出生人口素质
　　第十四章　节约保护资源，实现永续利用
　　第十五章　加强生态建设，保护和治理环境
　　第五篇改革开放
　　第十六章　推进改革，完善社会主义市场经济体制
　　第十七章　扩大对外开放，发展开放型经济
　　第六篇人民生活
　　第十八章　积极扩大就业，健全社会保障制度
　　第十九章　增加居民收入，提高人民生活水平
　　第七篇精神文明
　　第二十章　加强思想道德建设，形成共同理想和精神支柱
　　第二十一章　繁荣社会主义文化，提高文化生活质量
　　第八篇民主法制
　　第二十二章　加强民主政治建设，发展社会主义民主
　　第二十三章　依法治国，建设社会主义法治国家
　　第九篇国防建设
　　第二十四章　加强国防建设，保障国家安全
　　第十篇规划实施
　　第二十五章　改善宏观调控，保持经济稳定增长
　　第二十六章　创新实施机制，保障实现规划目标

　　序言

　　世纪之交，我国胜利实现了现代化建设的前两步战略目标，经济和社会全面发展，人民生活总体上达到了小康水平。从新世纪开始，我国将进入全面建设小康社会，加快推进社会主义现代化的新的发展阶段。
　　“九五”期间，面对错综复杂的国际国内经济环境，党中央、国务院审时度势，总揽全局，坚持用发展的办法解决前进中的问题，经过全国各族人民的共同努力，在有效治理通货膨胀，成功实现经济“软着陆”后，针对经济形势的变化，实行扩大内需的方针，果断实施积极的财政政策和稳健的货币政策，抑制了通货紧缩趋势，克服了亚洲金融危机和国内有效需求不足带来的困难，国民经济和社会发展取得巨大成就。经济运行质量与效益提高，综合国力进一步增强，国内生产总值年均增长8.3%。主要工农业产品产量位居世界前列，商品短缺状况基本结束。粮食等主要农产品生产能力明显提高，实现了农产品供给由长期短缺到总量基本平衡、丰年有余的历史性转变。工业结构调整取得积极进展，信息产业等高新技术产业迅速成长，淘汰落后和压缩过剩工业生产能力取得成效。服务业持续增长，就业岗位增加。基础设施建设成绩显著，“瓶颈”制约得到缓解。经济体制改革继续深化，社会主义市场经济体制初步建立。国有大中型企业改革和脱困的三年目标基本实现，调整和完善所有制结构取得重大进展，市场体系建设全面推进，宏观调控机制进一步健全。全方位对外开放格局基本形成，对外贸易和利用外资的规模扩大、结构改善、质量提高，开放型经济迅速发展。人民生活水平继续提高，消费结构改善，农村贫困人口的温饱问题基本解决。科教兴国战略和可持续发展战略有效实施，文化、卫生、体育等各项社会事业全面进步。廉政建设和反腐败斗争不断推进。社会主义精神文明建设和民主法制建设进一步加强。香港、澳门回到祖国怀抱，“一国两制”方针和基本法得到全面贯彻执行，祖国和平统一大业取得历史性进展。
　　经济和社会发展中也存在一些突出问题：一是产业结构不合理，地区发展不协调，城镇化水平低，国民经济整体素质不高，国际竞争力不强；二是科技、教育比较落后，科技创新能力弱，人才资源不足；三是水、石油等重要资源短缺，部分地区生态环境恶化；四是社会主义市场经济体制尚不完善，阻碍生产力发展的体制性因素仍然突出；五是人口压力大，就业矛盾尖锐，农民和城镇部分居民收入增长缓慢，收入差距拉大；六是一些领域市场经济秩序相当混乱，贪污腐败、奢侈浪费现象和形式主义、官僚主义作风还比较严重，有些地方社会治安状况不好等。对此，必须保持清醒的认识，认真解决。
　　今后五到十年，是我国经济和社会发展的重要时期，是进行经济结构战略性调整的重要时期，也是完善社会主义市场经济体制和扩大对外开放的重要时期。本纲要根据《[中共中央关于制定国民经济和社会发展第十个五年计划的建议](https://www.pkulaw.com/chl/37b66332069ffbc7bdfb.html)》编制，是战略性、宏观性、政策性的规划，是“十五”期间（2001年至2005年）国民经济和社会发展的宏伟蓝图，是全国各族人民共同奋斗的行动纲领。纲要主要阐述国家战略意图，明确政府工作重点，引导市场主体行为方向，所提出的产业发展方向和重点，是对市场主体的指导性意见，政府将运用经济政策等手段加以引导，在基础设施、科技和教育、生态环境、社会保障以及公共服务等领域提出的任务，政府将运用所掌握的公共资源，切实履行职责，努力完成。

　第一篇　指导方针和目标

　第一章　国民经济和社会发展的指导方针

　　经过二十多年的改革开放和发展，我国社会生产力、综合国力和人民生活水平都上了一个大台阶，市场供求关系、体制环境和对外经济关系发生了重大变化，为开始实施现代化建设第三步战略部署奠定了良好基础。进入二十一世纪，经济全球化趋势增强，科技革命迅猛发展，产业结构调整步伐加快，国际竞争更加激烈。世界经济形势的深刻变化和发展趋势，特别是加入世界贸易组织，将给我国带来新的发展机遇和严峻挑战。
　　面对改革开放和现代化建设新阶段的形势和任务，必须坚持党在社会主义初级阶段的基本理论、基本路线和基本纲领，进一步解放思想，实事求是，正确处理改革、发展、稳定的关系，确保社会稳定。“十五”期间要突出贯彻以下重要指导方针：
　　坚持把发展作为主题。发展是硬道理，是解决我国所有问题的关键。要认清世界经济发展趋势，增强紧迫感和忧患意识，坚持以经济建设为中心不动摇。继续实行扩大内需的方针，在坚持速度与效益相统一的基础上，抓住机遇，加快发展。
　　坚持把结构调整作为主线。我国已经进入必须通过结构调整才能促进经济发展的阶段。要以提高经济效益为中心，以提高国民经济的整体素质和国际竞争力、实现可持续发展为目标，积极主动、全方位地对经济结构进行战略性调整。要把调整产业结构与调整所有制结构、地区结构、城乡结构结合起来。坚持在发展中推进经济结构调整，在经济结构调整中保持快速发展。
　　坚持把改革开放和科技进步作为动力。改革开放是强国富民的必由之路，科技进步和创新是增强综合国力的决定性因素，经济发展和结构调整必须依靠体制创新和科技创新。要深化市场取向的改革，加快完善社会主义市场经济体制。坚定不移地扩大对外开放，在积极“引进来”的同时，实施“走出去”战略。加大实施科教兴国战略的力度，振兴科技，培养人才。
　　坚持把提高人民生活水平作为根本出发点。不断提高城乡居民的物质和文化生活水平，是社会主义的本质要求和发展经济的根本目的。要努力增加城乡居民特别是农民和城镇低收入者的收入。把不断增加农民收入放在经济工作的突出位置，千方百计促进农民收入较快增长。把扩大就业作为经济和社会发展的重要目标，实行有利于扩大就业的经济政策和社会政策。同时，要合理调节收入分配关系，加快健全社会保障制度。
　　坚持经济和社会协调发展。要把物质文明建设和精神文明建设作为统一的奋斗目标，把依法治国与以德治国结合起来，始终坚持两手抓、两手都要硬，切实加强社会主义精神文明和民主法制建设。要高度重视人口、资源、生态和环境问题，抓紧解决好粮食、水、石油等战略资源问题，把贯彻可持续发展战略提高到一个新的水平。

　第二章　国民经济和社会发展的主要目标

　　“十五”期间国民经济和社会发展的主要目标是：国民经济保持较快发展速度，经济结构战略性调整取得明显成效，经济增长质量和效益显著提高，为到2010年国内生产总值比2000年翻一番奠定坚实基础；国有企业建立现代企业制度取得重大进展，社会保障制度比较健全，完善社会主义市场经济体制迈出实质性步伐，在更大范围内和更深程度上参与国际经济合作与竞争；就业渠道拓宽，城乡居民收入持续增加，物质文化生活有较大改善，生态建设和环境保护得到加强；科技、教育加快发展，国民素质进一步提高，精神文明建设和民主法制建设取得明显进展。
　　“十五”期间宏观调控的主要预期目标是：经济增长速度预期为年均7%左右，到2005年按2000年价格计算的国内生产总值达到12.5万亿元左右，人均国内生产总值达到9400元。五年城镇新增就业和转移农业劳动力各达到4000万人，城镇登记失业率控制在5%左右。价格总水平基本稳定。国际收支基本平衡。
　　经济结构调整的主要预期目标是：产业结构优化升级，国际竞争力增强。2005年第一、二、三产业增加值占国内生产总值的比重分别为13%、51%和36%，从业人员占全社会从业人员的比重分别为44%、23%和33%。国民经济和社会信息化水平显著提高。基础设施进一步完善。地区间发展差距扩大的趋势得到有效控制。城镇化水平有所提高。
　　科技、教育发展的主要预期目标是：2005年全社会研究与开发经费占国内生产总值的比例提高到1.5%以上，科技创新能力增强，技术进步加快。各级各类教育加快发展，基本普及九年义务教育的成果进一步巩固，初中毛入学率达到90%以上，高中阶段教育和高等教育毛入学率力争分别达到60%左右和15%左右。
　　可持续发展的主要预期目标是：人口自然增长率控制在9‰以内，2005年全国总人口控制在13.3亿人以内。生态恶化趋势得到遏制，森林覆盖率提高到18.2%，城市建成区绿化覆盖率提高到35%。城乡环境质量改善，主要污染物排放总量比2000年减少10%。资源节约和保护取得明显成效。
　　提高人民生活水平的主要预期目标是：居民生活质量有较大提高，基本公共服务比较完善。城镇居民人均可支配收入和农村居民人均纯收入年均增长5%左右。2005年城镇居民人均住宅建筑面积增加到22平方米，全国有线电视入户率达到40%。城市医疗卫生服务水平和农村医疗服务设施继续改善，人民健康水平进一步提高。城乡文化、体育设施增加，覆盖面扩大，文化生活更加丰富。社会风气和社会秩序好转。

　第二篇　经济结构

　第三章　加强农业基础地位，促进农村经济全面发展

　　要始终把农业放在发展国民经济的首位，保证农业在提高整体素质和效益的基础上持续、稳定发展，农民收入较快增长。

　第一节　稳定粮食生产能力

　　严格执行基本农田保护制度，保持全国耕地总量动态平衡，确保到2005年全国耕地面积不低于12800万公顷。通过实施“种子工程”、完善农田水利配套设施、加强中低产田改造、调整商品粮基地建设的内容和布局等措施，稳定粮食生产能力。加大对粮食主产区的支持力度，保护粮食主产区生产粮食的积极性，鼓励与销区建立长期稳定的购销关系。确保粮食供求基本平衡。

　第二节　拓宽农民增收领域

　　通过加工转化、扩大出口等多种方式，解决粮食等农产品阶段性供过于求问题。调整农产品结构，发展多种经营，引导农户面向市场生产附加值高的产品。实行适度规模经营，推进农业机械化，提高劳动生产率，降低农产品生产成本。积极有序转移农村富余劳动力，引导农民更多地从事非农产业。建设一批有利于农民直接增收的项目，增加农民劳务收入。

　第三节　调整农业和农村经济结构

　　农业和农村经济结构调整，要面向市场，依靠科技，以农户和农业产业化经营企业为主体，不断向生产的广度和深度进军。以优化品种、提高质量、增加效益为中心，积极调整种植业作物结构、品种结构和品质结构，发展优质高产高效种植业。继续建设新疆优质棉基地、长江流域双低油菜基地及绿色食品基地。扩大饲料作物种植面积，加强草场建设，改良畜禽品种，加快发展畜牧业，2005年畜牧业产值占农业总产值的比重达到33%左右。加强渔业资源和渔业水域生态保护，积极发展水产养殖和远洋渔业。合理调整农业生产区域布局，发展特色农业，形成规模化、专业化的生产格局，提高商品率。加强对农产品出口的协调、服务和政策支持，继续建设农产品出口创汇基地，大力发展创汇农业。加快农产品加工技术和设备的引进开发，发展农产品销售、储运、保鲜等产业，提高农产品加工水平和效益。鼓励农业服务组织创新，培育经纪人队伍，全面发展农业社会化服务。加强农业质量标准体系、农产品质量检测检验体系和市场信息体系建设，加快制定或修订农业行业标准和重要农产品质量标准，推广采用国际标准，创建农产品标准化生产示范基地。支持引导乡镇企业推进结构调整、技术进步和体制创新，促进乡镇企业合理集聚、健康发展。
　　农业产业化经营是推进农业现代化的重要途径。鼓励采取“公司加农户”、“订单农业”等多种形式，大力推进农业产业化经营。支持农产品加工企业、销售企业和科研单位带动农户进入市场，与农户形成利益共享、风险共担的经营机制。采取财政、税收、信贷等方面的优惠政策，扶持一批重点龙头企业加快发展。

　第四节　加强农业和农村基础设施建设

　　搞好农田灌溉等农村中小型水利工程的维护和建设，继续对大型灌区进行以节水为中心的配套建设和改造，建设节水增效示范工程及旱作节水示范基地。支持农村公路建设，改善农村道路状况，完成国家级贫困县与国道、省道连接线的建设。发展沼气、节能灶等新能源和新型节能技术，加强农村能源综合建设。完成农村电网改造，实现城乡用电同网同价。努力解决无电地区的用电问题。加强农村通信基础设施建设，降低农村电信资费，实现城乡通话费同价。加强以产地批发市场为重点的农产品市场设施建设，完善农村市场体系。

　第五节　深化农村改革

　　加快农村土地制度法制化建设，长期稳定以家庭联产承包经营为基础、统分结合的双层经营体制。在长期稳定土地承包关系的基础上，鼓励有条件的地区积极探索土地经营权流转制度改革。根据粮食生产和流通的新情况，按产销区的不同特点，继续深化粮食流通体制市场取向的改革。完善粮食收购保护价、粮食储备和风险基金制度。全面推进农村税费改革，转变乡镇政府职能，适度撤并乡镇，精简乡镇机构和人员，减少村组干部补贴人数，切实减轻农民负担。改革农村金融体制，增加对农业的信贷投放，改进贷款方式，提高金融服务水平。要因地制宜地加快农村信用社管理体制改革，充分发挥农村信用社在农村金融服务中的主力军作用。深化农业科技体制改革，加大农业科技投入，支持农业科技创新，加强以农业科技成果推广为重点的科技服务体系建设，稳定基层农业技术推广队伍。

　第六节　扶持贫困地区发展

　　巩固“八七”扶贫攻坚成果，从根本上改变贫困地区面貌，是一项长期而艰巨的任务。要重点做好中西部的少数民族地区、革命老区、边疆地区和特困地区的扶贫工作，尽快使剩余贫困人口实现脱贫。坚持开发式扶贫，加大对贫困地区的财政转移支付力度，多方面增加扶贫资金投入，提高资金使用效益。加强贫困地区基础设施建设，加快发展教育、文化、卫生事业。扩大以工代赈规模，加强乡村道路、人畜饮水、基本农田、小型水利设施建设，改善贫困地区基本的生产和生活条件。对少数生存环境恶劣的贫困地区，要创造条件，易地开发。对返贫地区和人口继续给予支持。

　第四章　优化工业结构，增强国际竞争力

　　要坚持以市场为导向，以企业为主体，以技术进步为支撑，突出重点，有进有退，努力提高我国工业的整体素质和国际竞争力。工业改组改造要遵循市场经济规律，正确引导投资方向，依靠现有基础，防止盲目扩大规模和重复建设。坚持引进技术与自主创新相结合，先进技术与适用技术相结合。重点强化对传统产业的改造升级，进一步发挥劳动密集型产业的比较优势。积极发展高新技术产业和新兴产业，形成新的比较优势。以信息化带动工业化，发挥后发优势，实现社会生产力的跨越式发展。

　第一节　加快工业改组改造

　　加快转变工业增长方式，围绕增加品种、改善质量、节能降耗、防治污染和提高劳动生产率，鼓励采用高新技术和先进适用技术改造传统产业，带动产业结构优化升级。提高工业产品开发和深加工能力，优化产品结构。原材料工业，积极发展三大合成材料和精细化工产品、不锈钢和冷轧板、氧化铝、稀土深加工产品、新型干法水泥以及市场需求量大的高效化肥、药品及关键中间体等。轻纺工业，积极发展木浆、高档纸及纸板、新型家用电器、差别化纤维、产业用纺织品、高档面料、名牌服装及农产品深加工等。
　　研究制定振兴装备制造业的政策措施，依托重大工程，大力振兴装备制造业，提高先进技术装备的设计、制造和成套水平，增强能力。把发展数控机床、仪器仪表和基础零部件放到重要位置，努力提高质量和技术水平。支持发展大型燃气轮机、大型抽水蓄能机组、核电机组等新型高效发电设备，超高压直流输变电设备，大型冶金、化肥和石化成套设备，城市轨道交通设备，新型造纸和纺织机械等。发展农业机械、民用船舶和经济型轿车。提高汽车及关键零部件的制造水平。积极发展高效节能低排放车用发动机和混合动力系统。推进机电一体化。
　　积极发展建筑业，改进设计规范，提高设计、施工和监理水平，改革、整顿、规范建设市场，确保工程质量。推广使用新型建材。

　第二节　发展高技术产业

　　抓住世界科技革命迅猛发展的机遇，有重点地发展高技术产业，实现局部领域的突破和跨越式发展，逐步形成我国高技术产业的群体优势。建设一批重大高技术工程，主要是高速宽带信息网、深亚微米集成电路、生物技术工程、新型涡扇喷气支线客机、新型运载火箭等。促进数字化电子产品、新型显示器件、光电子材料与器件、现代中药、卫星应用等高技术的产业化，支持各行业发展高技术产品。完善风险投资机制，建立创业板股票市场，鼓励发展多种所有制的创新型中小企业。

　第三节　优化企业组织结构

　　按照专业化分工协作和规模经济原则，依靠优胜劣汰的市场机制和宏观调控，形成产业内适度集中、企业间充分竞争，大企业为主导、大中小企业协调发展的格局。通过上市、兼并、联合、重组等形式，形成一批拥有著名品牌和自主知识产权、主业突出、核心能力强的大公司和企业集团，提高产业集中度和产品开发能力。实行鼓励中小企业发展的政策，完善中小企业服务体系，促进中小企业向“专、精、特、新”的方向发展，提高与大企业的配套能力。
　　综合运用经济、法律和必要的行政手段，关闭产品质量低劣、浪费资源、污染严重、不具备安全生产条件的厂矿，淘汰落后设备、技术和工艺，压缩部分行业过剩和落后的生产能力。

　第四节　推进老工业基地结构调整

　　积极支持和促进东北等地的老工业基地改造和结构调整。充分发挥其基础雄厚、人才聚集的优势，结合国有经济布局调整，优化产业结构、企业组织结构和地区布局，形成新的优势产业和企业，有条件的地区要成为新的装备制造基地。积极稳妥地关闭资源枯竭的矿山，因地制宜地促进以资源开采为主的城市和大矿区发展接续产业和替代产业，研究探索矿山开发的新模式。

　第五节　以信息化带动工业化

　　通过微电子、计算机、网络技术的应用，推动产业研究开发和设计水平的提高以及工艺技术的变革；通过电子商务特别是企业间电子商务的应用，推动营销、运输和服务方式的变革，降低成本，扩大工业品市场规模；通过促进信息产品与传统产品的融合，以及信息技术在新产品中的广泛应用，增加产品的信息技术附加值。加速企业生产、经营管理的信息化进程。

　第五章　发展服务业，提高供给能力和水平

　　要以市场化、产业化和社会化为方向，增加供给，优化结构，拓宽领域，扩大就业，加快发展服务业。

　第一节　发展面向生活消费的服务业

　　深化城镇住房制度改革，落实住房分配货币化政策，发展以居民住宅为重点的房地产业和装修装饰业，规范发展物业管理业。加大旅游市场促销和新产品开发力度，加强旅游基础设施和配套设施建设，改善服务质量，促进旅游业成为新的经济增长点。优化配置和充实社区服务设施，鼓励创办各种便民利民的社区服务企业，壮大社区服务业。进一步发展商业零售业和餐饮业。理顺职业培训的管理体制，探索公办民助、民办公助、中外合作等多种办学方式，促进职业培训产业成长。积极发展面向生活消费的金融、保险服务。开发健康有益大众化的娱乐、健身项目，发展文化和体育产业。

　第二节　发展主要面向生产的服务业

　　积极引进新型业态和技术，推行连锁经营、物流配送、代理制、多式联运，改造提升传统流通业、运输业和邮政服务业。加快发展金融保险业，支持商业银行拓展信贷业务，拓宽保险资金运用渠道，提高保险公司承保能力和偿付能力，鼓励金融保险业务创新，改进服务。实现中介机构脱钩改制，确保其独立、客观、公正地执业，规范发展会计服务、法律服务、管理咨询、工程咨询等中介服务业。积极发展信息服务业特别是网络、信息技术应用咨询和数据库服务业。

　第三节　形成有利于服务业发展的体制环境

　　要转变观念，突破体制障碍，打破垄断，放宽市场准入。加快适宜产业化经营的社会事业的改革，实行政企（事）分开、企业与事业分开、营利性机构与非营利性机构分开。加快机关、学校、医院和企业事业单位后勤服务的社会化进程，逐步改制为独立法人企业。积极推进商业、外贸、供销、粮食等系统的改革，增强流通企业活力。拓宽服务业融资渠道。完善服务业标准，提高服务水平。

　第六章　加速发展信息产业，大力推进信息化

　　要按照应用主导、面向市场、网络共建、资源共享、技术创新、竞争开放的发展思路，努力实现我国信息产业的跨越式发展，加速推进信息化，提高信息产业在国民经济中的比重。

　第一节　广泛应用信息技术

　　加强信息资源开发，强化公共信息资源共享，推动信息技术在国民经济和社会发展各领域的广泛应用。实施一批信息化重大工程，推进政务、金融、外贸、广播电视、教育、科技、医疗卫生、社会保障和公用事业等领域的信息化进程。加快电子认证体系、现代支付系统和信用制度建设，大力发展电子商务。重视信息网络技术在农产品交易、农业技术推广等领域的应用。推动信息产业与有关文化产业结合。加强条码和代码等信息标准化基础工作。加快计算机和网络知识普及，各级各类学校要积极推广计算机及网络教育。制定国家信息系统标准，加强信息化法制建设，改革管理体制，创造有利于信息化的制度条件。

　第二节　建设信息基础设施

　　健全信息网络体系，提高网络容量和传输速度。大力发展高速宽带信息网，重点建设宽带接入网，适时建设第三代移动通信网。强化网络与信息安全保障体系建设。建设基础国情、公共信息资源、宏观经济数据库及其交换服务中心，完善地理空间信息系统。促进电信、电视、计算机三网融合。

　第三节　发展电子、信息产品制造业

　　加强先进信息技术的引进、消化吸收和创新，大力开发核心技术，增强计算机与网络产品、通信产品、数字视听产品和新型元器件等产品的制造能力，大力发展集成电路和软件产业，提高信息化装备和系统集成能力。

　第七章　加强基础设施建设，改善布局和结构

　　要优化结构，调整布局，提高工程质量，拓宽投资渠道，注重投资效益，把基础设施建设提高到一个新水平。

　第一节　加强水利建设

　　水利建设要全面规划，统筹兼顾，标本兼治，综合治理。坚持兴利除害结合，防洪抗旱并举，在加强防洪减灾的同时，把解决水资源不足和水污染问题放到更突出的位置。科学制定并积极实施全国水利建设总体规划和大江大河流域规划。重点加强大江大河大湖防洪工程体系建设和综合治理，对淤积严重的河湖进行整治和疏浚。加强以长江、黄河为重点的堤防建设。继续建设长江三峡、黄河小浪底等水利枢纽，兴建黄河沙坡头、嫩江尼尔基、淮河临淮岗、岷江紫坪铺、澧水皂市、右江百色、塔里木河及黑河整治等水利工程。加强蓄滞洪区安全建设，强化城市防洪，抓紧病险水库的除险加固。搞好水利设施配套建设和经营管理。加紧南水北调工程的前期工作，“十五”期间尽早开工建设。适时建设其他跨流域调水工程，采取多种方式缓解北方地区缺水矛盾。

　第二节　健全综合交通体系

　　交通建设要统筹规划，合理安排，扩大网络，优化结构，完善系统，推进改革，建立健全畅通、安全、便捷的现代综合运输体系。加快以“五纵七横”为重点的公路国道主干网建设，全面贯通“三纵两横”。起步建设西部公路的八条新通道，完善路网结构，提高路网通达深度。2005年公路通车里程达到160万公里左右，其中高速公路2.5万公里。建设改造“八纵八横”铁路主通道，扩大西部铁路网，加快铁路既有线技术改造，提高列车运行速度。建设青藏铁路、京沪高速铁路和西北、西南进出境铁路。2005年铁路营业里程达到7.5万公里。发展大城市轨道交通。加强沿海主枢纽港口大型集装箱运输系统、专业化散货运输系统及主要港口出海航道建设，建设上海国际航运中心。2005年沿海港口深水泊位达到800个。加强长江、珠江及京杭运河等水运主通道建设，积极发展内河航运。突出支线机场建设并向西部地区倾斜，完善枢纽机场和干线机场，提高空管技术装备水平。加强油气管道建设，初步形成管道运输网。充分发挥各种运输方式的优势，发展和完善城市间旅客快速运输、大城市旅客运输、集装箱运输、大宗物资运输和特种货物运输五大系统。以信息化、网络化为基础，加快智能型交通的发展。继续加强邮政基础设施建设。加快以政企分开为核心的交通运输管理体制和经营机制改革，铁路实行“网运分离”，民航机场和港口下放地方管理，航空运输企业实行重组，形成大集团。

　第三节　优化能源结构

　　能源建设要发挥资源优势，优化能源结构，提高利用效率，加强环境保护。以煤炭为基础能源，提高优质煤比重。推进大型煤矿改造，建设高产高效矿井，开发煤层气资源。加大洁净煤技术研究开发力度，通过示范广泛推广使用。实行油气并举，加快天然气勘探、开发和利用，统筹生产基地、输送管线和用气工程建设，引进国外天然气，提高天然气消费比重。开发燃料酒精等石油替代产品，采取措施节约石油消耗。加强石油资源勘探，合理开发石油资源，努力发展海洋石油。积极利用国外资源，建立海外石油、天然气供应基地，实行石油进口多元化。建立国家石油战略储备，维护国家能源安全。加强城乡电网建设和改造，建设西电东送的北、中、南三条大通道，推进全国联网。进一步调整电源结构，充分利用现有发电能力，积极发展水电、坑口大机组火电，压缩小火电，适度发展核电，鼓励热电联产和综合利用发电。开工建设龙滩、小湾、水布垭、构皮滩、三板溪、公伯峡、瀑布沟等大型水电站，抓紧长江上游溪洛渡或向家坝水电站开发的前期论证工作。在山西、陕西、内蒙古、宁夏、贵州、云南建设大型坑口电站。深化电力体制改革，逐步实行厂网分开、竞价上网，健全合理的电价形成机制。积极发展风能、太阳能、地热等新能源和可再生能源。推广能源节约和综合利用技术。

　第八章　实施西部大开发战略，促进地区协调发展

　　实施西部大开发战略，加快中西部地区发展，合理调整地区经济布局，促进地区经济协调发展。

　第一节　推进西部大开发

　　西部大开发要从实际出发，积极进取、量力而行，统筹规划、科学论证，突出重点、分步实施。力争用五到十年时间，使西部地区基础设施和生态环境建设有突破性进展，科技、教育有较大发展。要开拓新思路，采用新机制，着力改善投资环境，扩大对内对外开放，大力发展多种所有制经济，积极吸引社会资金和外资参与西部开发和建设。
　　加快水利、交通、通信、电网及城市基础设施建设，突出抓好西电东送、西气东输、节水和开发水资源等一批具有战略意义的重点工程。加强生态建设和环境保护，保护天然林资源，因地制宜实施坡耕地退耕还林还草，推进防沙治沙和草原保护，注意发挥生态的自我修复能力。巩固和加强西部地区的农业基础。发展有特色的农牧业、绿色食品、旅游、中草药及生物制药等，推进水电、石油天然气、有色金属、钾盐、磷矿等优势资源合理开发和深度加工，加快资源优势向经济优势的转化。坚持科教先行，重点发展义务教育，大力发展职业教育，积极发展高等教育，做好人才培养、使用和引进的工作。推广应用高新技术和先进适用技术，有重点地发展高新技术产业。依托亚欧大陆桥、长江水道、西南出海通道等交通干线及中心城市，以线串点，以点带面，实行重点开发，促进西陇海兰新线经济带、长江上游经济带和南（宁）贵（阳）昆（明）经济区的形成，提高城镇化水平。国家实行重点支持西部大开发的政策措施，增加对西部地区的财政转移支付和建设资金投入，并在对外开放、税收、土地、资源、人才等方面采取优惠政策。
　　依据[民族区域自治法](https://www.pkulaw.com/chl/7c03e99b1102a4a0bdfb.html)，支持民族自治地区落实自治权。加大支持力度，加快少数民族和民族地区经济与社会全面发展，重点支持少数民族地区的扶贫开发、牧区建设、民族特需用品生产、民族教育和民族文化事业发展。注意支持人口较少的少数民族的发展。促进西部边疆地区与周边国家和地区开展经济技术与贸易合作，逐步形成优势互补、互惠互利的国际区域合作新格局。

　第二节　加快中部地区发展

　　充分发挥中部地区承东启西、纵贯南北的区位优势和综合资源优势，加快发展步伐，提高工业化和城镇化水平。巩固农业的优势地位，大力发展农业产业化经营，形成区域性、专业化、大规模的农产品生产、流通和加工基地。加快调整工业结构，改造传统产业，建设能源基地，发展有竞争力的制造业和高新技术产业，大力发展服务业。搞好大江大河大湖治理，继续加强交通、通信、仓储等基础设施建设和生态环境建设。以长江、陇海、京广、京九、京哈等沿线地区为重点，壮大沿线城市规模，充实中心城市，积极培育新的经济增长点和经济带。

　第三节　提高东部地区的发展水平

　　东部地区要在体制创新、科技创新、对外开放和经济发展中继续走在前列，有条件的地方争取率先基本实现现代化。优化产业结构，优先发展高新技术产业、现代服务业和出口产业。发展外向型经济，广泛参与国际经济竞争。促进经济特区和浦东新区增创新优势，进一步发挥环渤海、长江三角洲、闽东南地区、珠江三角洲等经济区域在全国经济增长中的带动作用。发展黄河三角洲高效生态经济。东部地区要加强与中西部地区全方位的经济技术合作，支持和参与西部开发，更好地发挥对中西部地区的辐射带动作用。

　第四节　形成各具特色的区域经济

　　要打破行政分割，重塑市场经济条件下的新型地区经济关系。改变追求经济门类齐全的做法，发挥比较优势，发展有市场竞争优势的产业和产品，防止结构趋同。通过区域规划和政策，引导和调动地方的积极性，形成各具特色的区域经济，并先行在生态功能保护区、专业化农产品生产基地、旅游经济区等方面取得突破。

　第九章　实施城镇化战略，促进城乡共同进步

　　提高城镇化水平，转移农村人口，有利于农民增收致富，可以为经济发展提供广阔的市场和持久的动力，是优化城乡经济结构，促进国民经济良性循环和社会协调发展的重大措施。随着农业生产力水平的提高和工业化进程的加快，我国推进城镇化的条件已渐成熟，要不失时机地实施城镇化战略。

　第一节　形成合理的城镇体系

　　推进城镇化要遵循客观规律，与经济发展水平和市场发育程度相适应，循序渐进，走符合我国国情、大中小城市和小城镇协调发展的多样化城镇化道路，逐步形成合理的城镇体系。有重点地发展小城镇，积极发展中小城市，完善区域性中心城市功能，发挥大城市的辐射带动作用，引导城镇密集区有序发展。防止盲目扩大城市规模。要大力发展城镇经济，提高城镇吸纳就业的能力。加强城镇基础设施建设，健全城镇居住、公共服务和社区服务等功能。以创造良好的人居环境为中心，加强城镇生态建设和污染综合治理，改善城镇环境。加强城镇规划、设计、建设及综合管理，形成各具特色的城市风格，全面提高城镇管理水平。

　第二节　有重点地发展小城镇

　　发展小城镇是推进我国城镇化的重要途径。小城镇建设要合理布局，科学规划，体现特色，规模适度，注重实效。要把发展重点放到县城和部分基础条件好、发展潜力大的建制镇，使之尽快完善功能，集聚人口，发挥农村地域性经济、文化中心的作用。发展小城镇的关键在于繁荣小城镇经济，把引导农村各类企业合理集聚、完善农村市场体系、发展农业产业化经营和社会化服务等与小城镇建设结合起来。

　第三节　消除城镇化的体制和政策障碍

　　打破城乡分割体制，逐步建立市场经济体制下的新型城乡关系。改革城镇户籍制度，形成城乡人口有序流动的机制。取消对农村劳动力进入城镇就业的不合理限制，引导农村富余劳动力在城乡、地区间的有序流动。改革完善城镇用地制度，调整土地利用结构，盘活土地存量，在保护耕地和保障农民合法权益的前提下，妥善解决城镇建设用地。广辟投融资渠道，建立城镇建设投融资新体制，形成投资主体多元化格局。在政府引导下主要通过发挥市场机制作用建设小城镇，鼓励企业和城乡居民投资。科学制定设市、设镇标准，尽快形成符合市场经济体制和城镇化要求的行政管理体制。加强政策协调，改进城镇化的宏观管理。

　第三篇　科技、教育和人才

　第十章　推进科技进步和创新，提高持续发展能力

　　要面向经济建设，围绕结构调整，按照有所为、有所不为的方针，总体跟进，重点突破，发展高科技，实现产业化，提高科技持续创新能力，实现技术跨越式发展。力争在主要领域跟住世界先进水平，缩小差距；在有相对优势的部分领域，达到世界先进水平；在局部可跨越领域，实现突破。

　第一节　为产业升级提供技术支撑

　　以企业为技术创新主体，推进技术升级。加快开发能够推动结构升级和促进可持续发展的共性技术、关键技术和配套技术，重点在农产品加工及转化、节水农业、农作物新品种选育、装备制造、洁净煤、交通运输、纺织印染后整理、资源勘探开发、污水治理等方面取得进展，为产业结构调整特别是传统产业升级提供技术支撑。加强对引进技术的消化吸收和创新。

　第二节　推进高技术研究

　　积极推进高技术研究，在有相对优势或战略必争的关键领域取得突破，在一些关系国家经济命脉和安全的高技术领域，提高自主创新能力，努力实现产业化。重点攻克高速宽带网、高性能计算机、超大规模集成电路、大型应用软件、国家空间信息应用与服务等信息技术，生物芯片、遗传改良动植物、基因工程药物及疫苗等生物技术，特种功能材料、纳米材料、高性能结构材料等新材料技术，现代集成制造系统、机器人制造、飞机制造等先进制造技术和航空航天技术。

　第三节　加强基础研究和应用基础研究

　　加强数学、物理、化学、天文等基础学科重点领域的前沿性、交叉性研究和积累。加强应用基础研究，力争在基因组学、信息科学、纳米科学、生态科学、地球科学和空间科学等方面取得新进展。支持发展新兴学科、边缘学科和交叉学科，促进自然科学与社会科学的融合，推动管理科学发展。重视发展哲学社会科学和人文科学，调整学科布局，加强对重大理论和实践问题的研究，推进学科建设和理论创新。

　第四节　建设国家创新体系

　　要深化科技体制改革，形成符合市场经济要求和科技发展规律的新机制。优化科技资源配置，进一步解决科技与经济脱节问题，解决科研领域内的部门所有和单位分割等问题。建立企业技术创新体系，鼓励并引导企业建立研究开发机构，推动企业成为技术进步和创新的主体。建立为中小企业服务的技术创新支持系统，提高中小企业创新能力。加强产学研结合，建设一批行业共性技术的开发基地，鼓励应用开发型科研院所进入企业或改制为企业。积极推进社会公益性科研机构改革。建立国家知识创新体系，推进知识创新工程，促进大学与科研机构联合，形成一批具有国际影响的科研机构。加强科研基地和科研设施的联合共建，新建并重组部分国家重点实验室，建设重大科学工程，提高科研手段的现代化水平。加强中介服务体系建设，建立社会化的科技中介服务体系。扩大国际科技合作与交流，鼓励外资企业在我国设立研究开发机构。加大国家和全社会的科技投入，鼓励企业增加研究开发资金。加强知识产权保护。

　第十一章　加快教育发展，提高全民素质

　　教育是提高全民素质、培养人才的基础，要面向现代化、面向世界、面向未来，适度超前发展，走改革创新之路。

　第一节　发展各级各类教育

　　着力推进素质教育，重视培养创新精神和实践能力，促进学生德智体美全面发展。把加强基础教育放在重要位置，继续提高国民教育普及程度。基本普及九年义务教育的地区要巩固成果，提高水平，尚未普及的地区要进一步扩大九年义务教育的人口覆盖范围。重点推进西部贫困地区和少数民族地区的义务教育，实施贫困地区义务教育二期工程。扩大高中阶段教育规模，有步骤地在大中城市和经济发达地区普及高中阶段教育。采取多种形式，积极发展高等教育，扩大培养规模，保证教育质量。继续实施“211工程”，重点加强一批高水平大学和学科的建设。大力发展职业教育和职业培训，发展成人教育和其他继续教育，逐步形成大众化、社会化的终身教育体系。重视发展儿童早期教育。推行弹性学习制度，放宽入学年龄限制，允许分阶段完成学业。大力发展现代远程教育，提高教育现代化、信息化水平。加强教师队伍建设，提高教师思想和业务素质。加强和改进学校思想政治工作。根据世界经济与科技发展趋势及国内经济结构调整对人才的需求，大力调整教育结构和布局，并在优化学科和专业结构，更新教材，改革考试制度和教学方法等方面取得实质性进展。

　第二节　深化教育体制改革

　　加快办学体制改革，积极鼓励、支持和规范社会力量以多种形式办学，基本形成政府办学为主，公办学校和民办学校共同发展的格局。深化教育管理体制改革，进一步转变管理方式，依法落实高等学校办学自主权，推进高校后勤服务社会化改革。建立职业教育与普通教育相互沟通的教育体系。采取多种措施突破教育投入瓶颈，增加国家对教育的投入，加大中央和省级人民政府对义务教育的支持力度，加强县级人民政府对基础教育的统筹。完善政府对义务教育经费特别是教师工资的保障机制。深化与毕业生就业相关的劳动人事制度改革。健全奖学金、助学金和助学贷款等制度。

　第十二章　实施人才战略，壮大人才队伍

　　人才是最宝贵的资源，要把培养、吸引和用好人才作为一项重大的战略任务切实抓好，按照德才兼备的原则，培养数以亿计高素质的劳动者、数以千万计具有创新精神和创新能力的专门人才。

　第一节　开发人才资源

　　加快培养和选拔适应改革开放和现代化建设需要的各类人才。培养和造就坚持走有中国特色社会主义道路，有较高政治理论素养和开拓精神，掌握现代科学文化和管理知识，并经过实践考验的高素质领导人才队伍。培养具有科学素养、适应经济和社会发展需要的各类专业人才队伍，特别是具有世界前沿水平的学科带头人；具有创新精神和创业能力，适应国际竞争需要的企业家队伍；具有公仆意识，廉洁、勤政、高素质、专业化的公务员队伍；具有较高技术素质的技术工人队伍、农业产业化经营和农业科技队伍。注重在实践中培养和选拔人才。合理利用国外教育资源，拓宽高层次人才培养途径，积极吸引和聘用海外高级人才。继续实行支持留学、鼓励回国、来去自由的方针，鼓励留学人员回国工作或以适当方式为祖国服务。

　第二节　营造用好人才和吸引人才的良好环境

　　加快建立有利于优秀人才脱颖而出、人尽其才的有效机制，形成尊重知识、尊重人才、鼓励创业的社会氛围。进一步解放思想，转变观念，加大干部人事制度改革力度，实行公开招聘、竞争上岗制度，为各类人才特别是年轻优秀领导人才的成长、选拔和任用创造条件。优化人才的专业、年龄结构，促进人才在产业、地区、城乡间的合理流动和分布。完善对人才的激励机制和考核监督机制，为各类人才的创新、创业和发挥才能创造更为宽松的环境。建立和完善机制健全、运行规范、服务周到、指导监督有力的人才市场体系。完善人才使用、管理的法律法规。

　第四篇　人口、资源和环境

　第十三章　控制人口增长，提高出生人口素质

　　坚持计划生育的基本国策，稳定现行生育政策，保持低生育水平。重点做好农村特别是中西部地区农村的计划生育工作，加强对流动人口计划生育的管理。落实以宣传教育、避孕、经常性工作为主的方针，建立依法管理、村（居）民自治、优质服务、政策推动、综合治理的管理机制。继续实行党政领导责任制。加强人口与计划生育工作的法制建设。
　　提倡优生优育，大力开展计划生育服务，改善基层服务条件，开展生殖健康教育和服务。以预防农村地区高发先天性疾病为重点，明显降低出生缺陷发生率。建立和完善计划生育利益导向机制，对计划生育家庭实行奖励政策。开展农村独生子女户和双女户社会保险试点工作。

　第十四章　节约保护资源，实现永续利用

　　坚持资源开发与节约并举，把节约放在首位，依法保护和合理使用资源，提高资源利用率，实现永续利用。

　第一节　重视水资源的可持续利用

　　坚持开源节流并重，把节水放在突出位置。以提高用水效率为核心，全面推行各种节水技术和措施，发展节水型产业，建立节水型社会。城市建设和工农业布局要充分考虑水资源的承受能力。加大农业节水力度，减少灌溉用水损失，2005年灌溉用水有效利用系数达到0.45。按水资源分布调整工业布局，加快企业节水技术改造，2005年工业用水重复利用率达到60%。强化城市节水工作，强制淘汰浪费水的器具和设备，推广节水器具和设备。加强节水技术、设备的研究开发和节水设施的建设。加强规划与管理，搞好江河全流域水资源的合理配置，协调生活、生产和生态用水。加强江河源头的水源保护。积极开展人工增雨、污水处理回用、海水淡化。合理利用地下水资源，严格控制超采。多渠道开源，建设一批骨干水源工程，“十五”期间全国新增供水能力400亿立方米。加大水的管理体制改革力度，建立合理的水资源管理体制和水价形成机制。广泛开展节水宣传教育，提高全民节水意识。

　第二节　保护土地、森林、草原、海洋和矿产资源

　　坚持保护耕地的基本国策，实施土地利用总体规划，统筹安排各类建设用地，合理控制新增建设用地规模。加大城乡和工矿用地的整理、复垦力度。根据工业区、城镇密集区、专业化农产品生产基地、生态保护区等不同的土地需求，合理调整土地利用结构。强化森林防火、病虫害防治和采伐管理，完善林业行政执法管理体系和设施。加强草原保护，禁止乱采滥垦，严格实行草场禁牧期、禁牧区和轮牧制度，防止超载过牧。加大海洋资源调查、开发、保护和管理力度，加强海洋利用技术的研究开发，发展海洋产业。加强海域利用和管理，维护国家海洋权益。加强矿产资源勘探，严格整顿矿业秩序，对重要矿产资源实行强制性保护。深化矿产资源使用制度改革，规范和发展矿业权市场。推进资源综合利用技术研究开发，加强废旧物资回收利用，加快废弃物处理的产业化，促进废弃物转化为可用资源。

　第十五章　加强生态建设，保护和治理环境

　　要把改善生态、保护环境作为经济发展和提高人民生活质量的重要内容，加强生态建设，遏制生态恶化，加大环境保护和治理力度，提高城乡环境质量。

　第一节　加强生态建设

　　组织实施重点地区生态环境建设综合治理工程，长江上游、黄河上中游和东北内蒙古等地区的天然林保护工程，以及退耕还林还草工程。加强以京津风沙源和水源为重点的治理与保护，建设环京津生态圈。在过牧地区实行退牧，封地育草，实施“三化”草地治理工程。加快小流域治理，减少水土流失。推进黔桂滇岩溶地区石漠化综合治理。加快矿山生态恢复与治理。继续建设“三北”、沿海、珠江等防护林体系，加速营造速生丰产林和工业原料林。加快“绿色通道”建设，大力开展植树种草和城市绿化。加强自然保护区建设。保护珍稀、濒危生物资源和湿地资源，实施野生动物及其栖息地保护建设工程，恢复生态功能和生物多样性。“十五”期间，新增治理水土流失面积2500万公顷，治理“三化”草地面积1650万公顷。

　第二节　保护和治理环境

　　强化环境污染综合治理，使城乡特别是大中城市环境质量得到明显改善。抓紧治理水污染源，巩固“三河”、“三湖”水污染治理成果，启动长江上游、三峡库区、黄河中游和松花江流域水污染综合治理工程。加快城市污水处理设施建设，所有城市都要建设污水处理设施，2005年城市污水集中处理率达到45%。加强近岸海域水质保护，研究预防、控制和治理赤潮，抓好渤海环境综合整治和管理。加强大气污染防治，实施“两控区”和重点城市大气污染控制工程，2005年“两控区”二氧化硫排放量比2000年减少20%。推行垃圾无害化与危险废弃物集中处理。推行清洁生产，抓好重点行业的污染防治，控制和治理工业污染源，依法关闭污染严重、危害人民健康的企业。加强噪音污染治理。积极开展农村环境保护工作，防治不合理使用化肥、农药、农膜和超标污灌带来的化学污染及其他面源污染，保护农村饮用水水源。加强环境保护关键技术和工艺设备的研究开发，加快发展环保产业。完善环境标准和法规，修改不合理的污染物排放标准，健全环境监测体系，加强环境保护执法和监督。全面推行污水和垃圾处理收费制度。开展全民环保教育，提高全民环保意识，推行绿色消费方式。
　　加强防御各种灾害的安全网建设，建立灾害预报预防、灾情监测和紧急救援体系，提高防灾减灾能力。加强气象、地震、测绘等工作，提高服务能力和水平。积极参与全球环境与发展事务，履行义务，实行有利于减缓全球气候变化的政策措施。

　第五篇　改革开放

　第十六章　推进改革，完善社会主义市场经济体制

　　要大胆探索，勇于创新，突破影响生产力发展的体制性障碍，逐步完善社会主义市场经济体制。

　第一节　深化国有企业改革

　　进一步深化国有大中型企业改革，基本完成产权清晰、权责明确、政企分开、管理科学的现代企业制度的建设。健全责权统一、运转协调、有效制衡的公司法人治理结构。对国有大中型企业进行规范的公司制改革，除少数国家垄断经营的企业可改制为国有独资公司外，鼓励其他国有大中型企业通过规范上市、中外合资、相互参股等形式，逐步改制为多元持股的有限责任公司或股份有限公司。建立分工明确的国有资产管理、经营和监督体制，使国有资产出资人尽快到位，探索授权有条件的国有企业或国有资产经营公司行使出资人职能，强化对国有资产经营主体的外部监督。深化企业内部改革，强化科学管理，建立健全行之有效的激励机制和约束机制。进一步放开搞活国有中小企业，对国有小企业继续采取改组、联合、兼并、租赁、承包经营和股份合作制、出售等多种形式，进行产权制度和经营机制改革。完善市场退出机制，积极疏通和逐步规范企业特别是上市亏损公司退出市场的通道。继续执行现行的国有企业兼并破产政策，对长期亏损、资不抵债、扭亏无望的企业依法实施破产。鼓励非国有企业、个人和境外投资者参与国有企业改制，推动非上市国有企业股权结构的调整和股权交易，形成混合所有制企业。规范国有企业改制行为，完善有关政策规定，强化对国有资产产权交易的监督。

　第二节　调整和完善所有制结构

　　要坚持公有制为主体、多种所有制经济共同发展的基本经济制度。积极探索各种有效方式，有进有退，有所为有所不为，加快国有经济布局的战略性调整。发挥国有经济在国民经济中的主导作用，发展多种形式的集体经济，支持、鼓励和引导私营、个体企业健康发展。关系国家安全和经济命脉的重要企业要由国家控股。鼓励在高新技术产业、中介服务等领域创建无限责任和有限责任相结合的公司。取消一切限制企业和社会投资的不合理规定，在市场准入、土地使用、信贷、税收、上市融资、进出口等各方面，对不同所有制企业实行同等待遇。凡是对外资开放的领域，内资均可进入。依法保护各种所有制企业的合法权益。

　第三节　健全市场体系

　　进一步开放市场，放开价格，继续发展商品市场，重点培育和发展要素市场，建立和完善全国统一、公平竞争、规范有序的市场体系。引入竞争机制，加快电力、铁路、民航、通信、公用事业等垄断行业管理体制改革。破除地方封锁，反对地方保护主义，废除阻碍统一市场形成的各种规定。积极发展大宗商品批发市场，稳步发展期货市场。健全统一、安全、高效、开放的国债市场。疏通储蓄转化为投资的渠道，积极稳妥地发展多种融资方式。加强对证券市场的监管，提高证券市场的透明度，规范和发展证券市场，逐步提高直接融资比重。加快发展企业债券市场，发展产业投资基金。积极培育证券投资基金、养老基金和保险基金等机构投资者。发展票据市场，完善外汇市场，建立黄金交易市场。坚持城乡统筹就业的改革方向，推动城乡劳动力市场逐步一体化。深化土地使用制度改革，全面推行经营性用地招标拍卖制度，规范土地一级市场，活跃房地产二级市场，发展住房租赁市场。改革价格管理体制，进一步完善市场形成价格的机制。建立健全政府价格决策听证等制度，提高政府定价的科学性、时效性和透明度。

　第四节　深化金融体制改革

　　建立和完善金融组织体系、市场体系、监管体系和调控体系。对国有独资商业银行进行综合改革，有条件的国有独资商业银行可以改组为国家控股的股份制商业银行，建立风险防范机制，提高竞争能力。在健全监管体系的基础上，规范发展中小金融机构。完善和发挥政策性银行的功能。推进保险市场主体多元化。完善金融机构内部治理结构，形成严格约束与有效激励相统一的经营机制，完善稳健的会计制度，提高金融资产质量。强化金融监管，防范和化解金融风险。完善金融分业监管构架，加强监管机构之间的协调配合。完善以间接调控为主的中央银行调控体系，积极稳妥地发展货币市场，扩大公开市场业务，稳步推进利率市场化改革。改进人民币汇率形成机制，完善以市场供求为基础的、有管理的浮动汇率制度。

　第五节　改革投资体制

　　确立企业在竞争性领域的投资主体地位，基本形成企业自主决策、自担风险，银行独立审贷，政府宏观调控的新的投资体制。按照“谁投资、谁决策、谁受益、谁承担风险”的原则，改革投资管理方式，减少对企业和社会投资的行政性审批。对企业出资建设国家非限制类项目，实行登记备案制。建立以政策引导、信息发布等间接调控手段为主的投资宏观调控体系。全面实行建设项目法人责任制、工程监理制、招标投标制、合同管理制。建立严格的政府投资项目管理机制，完善国家重大投资项目稽察制度。推动投资中介组织的市场化改革。

　第六节　深化财税体制改革

　　积极稳妥地推进税费改革，清理整顿行政事业性收费和政府性基金，建立政府统一预算。健全税收制度，改革生产型增值税税制，完善消费税和营业税，逐步统一内外资企业所得税，建立综合与分类相结合的个人所得税制度，适时开征社会保障税和遗产税，完善地方税税制。依法加强税收征管，打击偷、漏、骗税的行为，清缴欠税，严禁越权减、免、退税。
　　增强预算的透明度和约束力。实行部门预算，逐步以“零基预算”取代“基数预算”。改革国库制度，建立以国库单一账户体系为基础的现代国库集中收付制度。规范政府采购行为，全面推行政府采购制度。调整财政支出结构，压缩竞争性领域的支出。合理界定中央和地方政府的事权范围，完善分税制和转移支付制度，加强财政再分配功能。加强审计监督，严肃财经纪律。强化财政监督，防范财政风险，合理控制财政赤字和政府发债规模。保持财政收入稳定增长，提高国家财政收入占国内生产总值的比重和中央财政收入占全国财政收入的比重。逐步建立适应社会主义市场经济要求的公共财政框架，建设稳固、平衡、强大的国家财政。

　第七节　推进行政管理体制和政府机构改革

　　继续推进行政管理体制和政府机构改革，建立廉洁高效、运转协调、行为规范的行政管理体制，推进政府决策的民主化、科学化。按照发展社会主义市场经济的要求，进一步转变政府职能，集中精力搞好宏观调控和创造良好的市场环境，不直接干预企业正常的生产经营活动。加快政府审批制度改革，大幅度减少行政性审批，规范审批行为，强化监督机制。发挥商会、行业协会等中介组织的作用。继续改革和精简政府机构，解决部门职责不清、管理层次多、办事效率不高等问题。完善公务员制度。

　第十七章　扩大对外开放，发展开放型经济

　　要以更加积极的姿态，抓住机遇，迎接挑战，趋利避害，做好加入世界贸易组织的准备和过渡期的各项工作，不断提高企业竞争能力，进一步推动全方位、多层次、宽领域的对外开放。

　第一节　提高对外开放水平

　　根据我国经济和社会发展、结构调整的需要以及加入世界贸易组织的承诺，逐步降低关税，有步骤地开放银行、保险、电信、外贸、内贸、旅游等服务领域。对外商投资企业逐步实行国民待遇，制定统一、规范、透明的投资准入政策，除关系国家安全和经济命脉的重要行业或企业必须由国家控股外，取消对其他企业的股比限制。适应加入世界贸易组织的要求，抓紧制定和完善相关涉外法律法规，提高涉外经济工作依法行政的水平。深化改革，建立健全符合国际通行规则和我国国情的对外经济贸易体制。实行外贸经营资格登记注册制度，逐步实现对各类企业进出口贸易的放开经营。建立和完善对外经济贸易促进和服务体系，发挥中介组织的作用。
　　完善反倾销、反补贴及保障措施等手段。加强对外国反倾销、反补贴的磋商和应诉，维护我国企业的合法权益。借鉴和采用国际标准，推进技术性贸易措施体系建设，有效实施健康、安全、卫生、环保等方面的检验检疫和疫情监控，防止有害物质和生物入境。积极参与多边贸易体系和国际区域经济合作。加强和改善双边经贸关系，进一步加强与发展中国家的经济技术合作与交流。积极参与国际经济、贸易、金融等方面的规则制定，维护我国作为发展中国家的正当权益。

　第二节　积极发展对外贸易

　　更好地实施以质取胜、市场多元化和科技兴贸战略，努力扩大货物和服务出口，2005年货物进出口总额达到6800亿美元。积极开拓新的出口市场，优化出口商品结构和市场结构，继续扩大大宗传统产品和劳动密集型工业制成品出口，不断提高其技术含量和附加值，增加高新技术产品和高附加值产品出口，2005年机电产品出口比重提高到50%左右。加强加工贸易管理，提高加工贸易的增值率，扩大加工贸易出口。继续规范发展边境贸易。大力发展承包工程、设计咨询、技术转让、国际旅游、国际运输、航天发射、教育文化和金融保险等领域的服务贸易出口，逐步缩小服务贸易逆差。积极引进先进技术、关键设备，努力实现大宗产品和重要资源进口来源多元化。加强口岸查验、税务、金融、保险和运输等对进出口贸易的服务功能。严厉打击走私、逃套汇、骗取出口退税等不法行为，维护对外经济贸易秩序。

　第三节　积极合理有效地利用外资

　　把吸收外商直接投资作为利用外资的重点，完善利用外资政策，改善投资环境，扩大利用外资规模，提高利用外资质量。鼓励外商特别是跨国公司参与国有企业的改组改造，投资高新技术产业和出口型产业，促进我国产业结构调整和技术水平的提高。鼓励有条件的企业对外发行股票，积极探索采用收购、兼并、风险投资、投资基金等各种方式，促进利用外资和国有企业产权制度改革。鼓励和促进中外中小企业之间的合作。不断提高服务业利用外资的比重。继续发挥经济特区、浦东新区及其他沿海地区的优势，提高利用外资水平。采取鼓励政策，引导外资更多地投向中西部地区，特别是中西部地区的老工业基地改造、基础设施建设、生态建设和环境保护、矿产和旅游资源开发、优势产业发展等。继续合理利用国际金融组织和外国政府贷款，并更多地用于中西部地区。努力降低筹资成本，用好银团贷款、融资租赁、出口信贷等国际商业贷款。强化外债全口径管理，保持合理的外债规模，健全责权利统一的借用还机制，加强外债监测和短期资本监管，优化债务结构，防范债务风险。

　第四节　实施“走出去”战略

　　鼓励能够发挥我国比较优势的对外投资，扩大国际经济技术合作的领域、途径和方式。继续发展对外承包工程和劳务合作，鼓励有竞争优势的企业开展境外加工贸易，带动产品、服务和技术出口。支持到境外合作开发国内短缺资源，促进国内产业结构调整和资源置换。鼓励企业利用国外智力资源，在境外设立研究开发机构和设计中心。支持有实力的企业跨国经营，实现国际化发展。健全对境外投资的服务体系，在金融、保险、外汇、财税、人才、法律、信息服务、出入境管理等方面，为实施“走出去”战略创造条件。完善境外投资企业的法人治理结构和内部约束机制，规范对外投资的监管。

　第六篇　人民生活

　第十八章　积极扩大就业，健全社会保障制度

　　扩大就业是促进经济发展和维护社会稳定的重要保证，也是宏观调控的一项重要内容，要努力开拓就业渠道，扩大就业。完善的社会保障制度是社会主义市场经济体制的重要支柱，关系改革、发展、稳定的全局，“十五”期间要基本建成独立于企业事业单位之外、资金来源多元化、保障制度规范化、管理服务社会化的社会保障体系。

　第一节　积极扩大就业

　　保持较快的经济增长速度，创造更多的就业岗位。制定和落实优惠政策，发展具有比较优势的劳动密集型产业，发展就业容量大的服务企业、中小企业和非公有制企业。加大在职和再就业培训力度，提高从业人员的职业技能，增强失业人员的就业和创业能力。引导劳动者转变就业观念，采取非全日制就业、季节性就业等灵活多样的就业形式，提倡自主就业。努力开拓国际劳务市场，扩大劳务输出。全面推行劳动预备制度和职业资格证书制度，严格执行离退休制度。加强劳动力市场建设，完善就业服务体系，促进劳动力合理流动。
　　高度重视安全生产，改革安全生产管理体制，加快安全生产法制建设。抓好重点行业的安全整顿，加强安全监察，明显减少重大、特大事故，切实保障劳动者生命安全。

　第二节　健全社会保险制度

　　依法扩大养老保险实施范围，继续完善社会统筹与个人账户相结合的城镇职工基本养老保险制度，在保证离退休人员基本养老金支付的基础上，实现社会统筹基金与个人账户基金的分账管理，确保个人账户的有效积累。按社会统筹和个人账户相结合的模式，全面推行城镇职工基本医疗保险制度，保障职工基本医疗需求。同步推进医疗保险制度、医疗机构和药品生产流通体制改革，建立医疗费用的分担机制、医疗机构的竞争机制和药品流通的市场运行机制。进一步完善失业保险制度，在试点的基础上逐步把国有企业下岗职工基本生活保障纳入失业保险，扩大失业保险覆盖范围。完善工伤保险和生育保险制度，逐步扩大覆盖面。适时改革并完善机关事业单位职工基本养老保险制度。鼓励有条件的用人单位为职工建立企业年金和补充医疗保险，同时发挥商业保险对社会保障体系的补充作用。
　　建立可靠、稳定的社会保障资金筹措机制，通过加大征缴力度、调整财政支出结构、变现部分国有资产、扩大彩票发行规模等方式多渠道筹集社会保障资金。积极探索并建立规范的社会保障资金投资运营的方式，实现保值增值。提高社会保障资金的管理水平，加强社会保障行政监督和社会监督，推进社会保障管理和服务的社会化。加快社会保障立法步伐。

　第三节　发展其他社会保障事业

　　在健全社会保险制度的同时，继续发展社会福利、社会救济、优抚安置和社会互助等社会保障事业，推进社会福利的社会化进程。完善城市居民最低生活保障制度，扩大覆盖范围，根据经济发展水平和财政承受能力，逐步提高城市贫困人口救济补助标准。建立社会医疗救助制度。发展慈善事业，加强对捐助资金使用的监管。重视人口老龄化趋势，鼓励家庭养老。加强老年人服务设施建设，发展老龄事业和产业。贯彻落实妇女、儿童发展纲要，切实保障妇女、未成年人的合法权益。加强残疾人事业，帮助残疾人康复、就学和就业，创造残疾人平等参与社会生活的条件。

　第十九章　增加居民收入，提高人民生活水平

　　在经济发展的同时，不断增加城乡居民特别是农民和城镇低收入者的收入，努力实现城乡居民收入增长的预期目标。进一步提高城乡居民的消费水平，改善消费环境，优化消费结构。增加对公共服务的投入，为实现人人享有基本公共服务创造条件。

　第一节　深化收入分配制度改革

　　坚持效率优先、兼顾公平的原则，实行按劳分配为主体、多种分配方式并存的分配制度，把按劳分配与按生产要素分配结合起来。发挥市场机制对初次分配的基础性调节作用，健全劳动力价格的市场形成机制，推行职工工资集体协商制度，规范全社会工资性收入的结构和支付行为。建立健全与经济发展水平相适应的最低工资保障制度和最低工资标准调整机制。逐步提高国家机关和事业单位人员的工资待遇。改进和完善国有企业收入分配制度。建立健全收入分配的激励机制，要提高国有企业高层管理人员、技术人员的工资报酬，充分体现他们的劳动价值，可以试行年薪制。对国有上市公司负责人和技术骨干还可以试行期权制。同时要建立严格的约束、监督和制裁制度。鼓励资本、技术等生产要素参与收益分配。规范社会分配秩序，加强对垄断行业收入分配的监管，对依靠垄断地位获取的不合理收入进行调节。强化税收对收入分配的调节功能。保护合法收入，整顿不合理收入，调节过高收入，取缔非法收入，防止收入分配两极分化。

　第二节　拓宽消费领域

　　在提高居民吃穿用等基本消费水平的基础上，重点改善居民居住和出行条件。完善住房公积金制度，加大以经济适用住房为重点的住房建设力度，建立廉租房供应保障体系。积极发展城乡公共交通，鼓励轿车进入家庭。降低信息服务价格，刺激居民信息消费。提高计算机普及率，2005年达到4%以上。推行职工带薪休假制度，合理调整居民工作和闲暇时间，扩大服务供给，鼓励居民服务性消费。加强城乡供水、供电、供气、供热、信息、环境等基础设施建设，扩大农村基础设施的覆盖面，为改善城乡居民生活质量创造条件。建立个人信用制度，扩大消费信贷规模。

　第三节　发展卫生、体育事业

　　改革和完善卫生服务、医疗保障和卫生监督体系，发展基本医疗、预防保健、康复医疗。实施区域卫生规划，调整和优化卫生资源配置。高度重视卫生防病工作，加强对多发病、地方病、职业病和传染病的预防与控制。大力发展中医药，促进中西医结合。健全农村初级卫生保健服务体系，重点加强农村卫生基础设施建设，因地制宜发展合作医疗，努力解决农民基本卫生医疗问题。加强公共体育设施建设，开展全民健身运动，健全国民体质监测系统。发展竞技体育。

　第四节　推进社区建设

　　推进社区建设是新时期我国经济和社会发展的重要内容。要坚持政府指导与社会参与相结合，建立与社会主义市场经济体制相适应的社区管理体制和运行机制。加强社区组织和队伍建设，扩充社区管理职能，承接企业事业单位、政府机关剥离的部分社会职能和服务职能。以拓展社区服务为龙头，不断丰富社区建设的内容，发展社区卫生，繁荣社区文化，美化社区环境，加强社区治安，完善社区功能。努力建设管理有序、服务完善、环境优美、治安良好、生活便利、人际关系和谐的新型现代化社区。

　第七篇　精神文明

　第二十章　加强思想道德建设，形成共同理想和精神支柱

　　把依法治国与以德治国结合起来，大力推进社会主义精神文明建设，满足人民群众日益增长的多方面的精神文化需求，全面提高国民素质，增强民族凝聚力，为改革开放和现代化建设提供强大的思想保证、精神动力和智力支持。
　　以理想信念教育为核心，坚持不懈地进行党的基本理论、基本路线、基本纲领的教育，巩固和加强马克思主义的指导地位，用马列主义、毛泽东思想、邓小平理论教育人民，弘扬爱国主义、集体主义、社会主义精神，在全社会形成建设有中国特色社会主义的共同理想和精神支柱。发扬自力更生、艰苦奋斗的创业精神，提倡勤俭建国，反对奢侈浪费，形成全民族奋发向上的强大动力。深入开展“三个代表”重要思想的教育，弘扬讲学习、讲政治、讲正气的风气。大力倡导社会公德、家庭美德和职业道德，特别是加强青少年的思想政治、道德品质、心理健康和法制教育，努力建立适应社会主义市场经济发展的思想道德体系。创造青少年健康成长的社会环境。加强科学知识、科学方法、科学思想、科学精神的宣传教育和普及，反对封建迷信。以提高公民素质、城乡文明程度和居民生活质量为目标，大力开展群众性精神文明创建活动，倡导科学文明健康的生活方式。重视对社会思潮及其表现形式的研究和引导。加强对新闻舆论、国民教育、社会文化、休闲娱乐等各种思想文化阵地尤其是新闻网站的建设和管理。加强烈士陵园等爱国主义教育基地的保护和建设。

　第二十一章　繁荣社会主义文化，提高文化生活质量

　　坚持为人民服务、为社会主义服务的方向和百花齐放、百家争鸣的方针，以繁荣社会主义文化为中心，弘扬民族优秀文化，吸收外国文化有益成果，抵制不良文化，提高全社会的文化生活质量。
　　把握时代精神，坚持正确方向，发展新闻出版、广播影视等各项事业。加强文艺理论研究，繁荣文学艺术，努力提高精神产品的质量，生产出更多的无愧于时代和人民的优秀作品。加强图书馆、博物馆、文化馆、科技馆、档案馆和青少年活动场所等文化设施建设。努力巩固和拓展社会主义文化阵地，形成健康向上的舆论环境、文明和谐的社会氛围和丰富多彩的文化生活。坚持把社会效益放在首位、社会效益和经济效益相统一的原则，深化文化、广播影视、新闻出版体制改革，建立科学合理、灵活高效的管理体制和文化产品生产经营机制。继续实行支持文化事业发展的有关政策，增加对重要新闻媒体和公益文化事业的投入。加强民族文化遗产保护。积极开展对外文化交流。完善文化产业政策，加强文化市场建设和管理，推动有关文化产业发展。

　第八篇　民主法制

　第二十二章　加强民主政治建设，发展社会主义民主

　　适应经济体制改革和现代化建设的要求，继续推进政治体制改革，加强民主政治建设，发展社会主义民主。
　　继续加强人民代表大会的立法和监督工作，密切人大代表同人民的联系。充分发挥人民政协政治协商、民主监督、参政议政的作用，支持各民主党派工作。各级政府都要自觉接受同级人大的监督，主动听取政协和各社会团体的意见。实行民主选举、民主决策、民主管理和民主监督，保证人民依法享有广泛的权利和自由，尊重和保障人权。加强城乡基层政权机关和群众性自治组织建设，扩大公民有序的政治参与，引导人民群众依法参与经济、文化和社会事务的管理。做好信访工作。完善村民自治，加强社区民主建设，坚持和完善以职工代表大会为基本形式的企业民主管理制度，实行政务、厂务、村务公开。发挥工会、共青团、妇联等群众组织的桥梁和纽带作用。坚持和完善民族区域自治制度，落实党的民族政策，发展平等、团结、互助的社会主义新型民族关系，促进各民族共同繁荣和进步。全面贯彻党的宗教政策，保障公民宗教信仰自由，依法管理宗教事务，积极引导宗教与社会主义社会相适应。贯彻党的侨务政策，广泛发展爱国统一战线。

　第二十三章　依法治国，建设社会主义法治国家

　　坚持有法可依、有法必依、执法必严、违法必究的原则，从立法、司法、行政执法、法律监督、法律服务和普及法律知识等方面全面推进法制建设。
　　提高立法质量和效率，重点建立和完善适应社会主义市场经济体制的法律体系，规范市场经济条件下的财产关系、信用关系和契约关系。推进规范国家权力运行、社会保障和社会中介组织等方面的法律法规建设。推进司法改革，完善司法体制，健全侦查、检察、审判、执行等制度，强化司法保障和法律监督，支持依法独立行使审判权和检察权。依法办案，反对地方保护主义，严格执法，公正司法。推进政府工作法制化，依法行政，从严治政。加快推行执法责任制、评议考核制，提高行政执法水平。健全依法行使权力的制约机制，加强对权力运行的民主监督、群众监督和舆论监督。加快廉政建设法制化步伐，深入开展反腐败斗争，严肃查办违法违纪案件。推进国家各项事业的依法管理，提高社会管理的法制化水平。深入开展法制宣传教育，进一步提高全体公民首先是各级领导干部的法律意识和法律素质。建立法律援助体系。加大政法基础设施建设力度，建设高素质的政法队伍。
　　认真研究社会稳定面临的新情况新问题，正确处理新时期人民内部矛盾，确保社会稳定。依法打击民族分裂活动、宗教极端势力、暴力恐怖活动、邪教和利用宗教进行非法活动。切实把社会治安综合治理措施落实到基层，严厉打击危害国家安全和社会治安的刑事犯罪活动和各种恶势力，扫除黄赌毒等社会丑恶现象。加强治安防范工作，提高对突发性事件的快速反应和处理能力。

　第九篇　国防建设

　第二十四章　加强国防建设，保障国家安全

　　强大的国防是国家安全和现代化建设的基本保证。在集中力量发展国民经济的同时，要积极推进国防建设，增强国防实力。
　　军队建设是国防建设的主体。要加强军队党的建设和思想政治工作，确保党对军队的绝对领导。加强人民解放军的革命化、现代化、正规化建设。贯彻积极防御的军事战略方针，坚持质量建军，科技强军，勤俭建军，依法治军，积极改革创新。加快军队向质量效能型和科技密集型转变，走有中国特色的精兵之路，提高现代技术特别是高技术条件下防卫作战的能力，做好应付各种复杂情况的准备。加强国防动员体系建设，增强国防后备力量，提高平战转换能力。深化后勤保障改革，提高保障能力。完善国防立法。深入开展国防教育，提高全民国防意识。进一步密切军政军民关系，做好“双拥”工作。
　　国防科技工业是国家战略性产业。要通过加速调整、改革和发展，坚持军民结合，寓军于民，大力协同，自主创新，建立适应国防建设和市场经济要求的新型国防科技工业体制。发展高新技术武器装备，努力提高现代化水平。发展军民两用技术，促进军工技术的和平利用。
　　实现祖国统一是中华民族的根本利益。要坚持“和平统一、一国两制”的基本方针，坚决打击“***”分裂势力，早日完成祖国统一大业。

　第十篇　规划实施

　第二十五章　改善宏观调控，保持经济稳定增长

　　要综合运用计划、财政、金融等手段，发挥价格、税收、利率、汇率等杠杆的作用，加强和改善宏观调控，为规划实施创造良好的宏观经济环境。
　　宏观调控总的要求是：把扩大国内需求作为经济发展的基本立足点和长期战略方针，努力扩大对外贸易，保持经济总量平衡，引导和促进经济结构优化升级，实现经济稳定增长，扩大就业规模，保持价格总水平基本稳定和国际收支基本平衡。
　　宏观调控政策的基本取向是：近期要继续实行积极的财政政策，带动企业和社会投资，促进消费。实行稳健的货币政策，灵活运用货币政策工具，适时调节货币供应量，维护人民币币值和汇率基本稳定，支持经济结构调整和经济增长。实行与财政、货币政策相配套的价格政策，稳定价格总水平，警惕和防止通货膨胀。实行鼓励消费的政策，促进消费需求较快增长，使居民消费率提高到50%左右。围绕结构调整，引导投资方向，促进固定资产投资特别是企业和社会投资较快增长，全社会固定资产投资率调控在35%左右。实行支持出口的政策，发挥出口需求对经济增长的拉动作用。宏观调控政策的力度，要根据宏观经济运行态势，进行适时适度的调节。

　第二十六章　创新实施机制，保障实现规划目标

　　适应社会主义市场经济体制的要求，必须创新规划实施机制。政府要切实转变职能，正确履行职责，为规划实施创造良好的体制、政策、市场等环境。
　　推进体制改革。要在正确处理改革、发展、稳定关系的基础上，加快推进本纲要提出的各项改革，分步实现改革目标。在国家宏观调控下，充分发挥市场配置资源的基础性作用，激发市场主体的积极性和创造性。
　　制定经济政策。围绕经济结构的战略性调整，制定和实施产业发展政策、地区发展政策、城镇化政策、投资政策等，引导资源配置方向，提高资源配置效率。调整收入分配政策，逐步缩小收入差距。制定消费政策，提高居民生活质量。适应加入世界贸易组织的新形势，完善对外经济政策，发挥比较优势，保障国家经济安全。合理配置财政性资金、政策性金融、国家统借内外债、国家外汇储备以及国家物资储备、国有土地和国有资产存量等公共资源，发挥政府对资源配置的引导和带动作用。
　　整治市场秩序。制定和完善维护市场秩序的规则，反对垄断和不正当竞争，保证市场竞争机制的有效运行。完善市场监督机制，运用现代科技手段，加大监管力度。严厉打击制售假冒伪劣产品的行为，保护生产者和消费者合法权益。禁止价格欺诈、价格垄断、价格歧视、低价倾销等不正当价格行为。加强对建设项目的监管，加大稽察力度，严肃查处工程建设领域虚假招投标、虚假合同等违法行为。加强质量与标准化、计量基础工作，建立健全质量标准和计量检测体系，强化质量技术监督。严格资质认定，强化行业自律，提高中介组织的质量。在全社会强化信用意识，整肃信用秩序，建立严格的信用制度，依法惩处经济欺诈、逃废债务、不履行合约、侵犯知识产权等不法行为。严禁经济工作中的弄虚作假，保证基础信息、基础统计的真实性、准确性。
　　提供公共服务。政府配置资源的重点要逐步转向为全社会提供充足优质的公共产品和服务。主要是促进教育发展，加强基础研究和重大应用研究，健全社会保障体系，完善公共基础设施，发展公共事业，保护国土资源，加强生态建设和环境保护，维护社会治安，确保国家安全等。合理划分中央与地方的事权，充分发挥地方政府的积极性。
　　集中力量办大事。在组织实施城镇化、科技和教育、人口就业和社会保障、生态环境、水利、能源、交通、西部开发、信息化、加入世界贸易组织等重点专项规划时，中央政府将在关系全局的关键领域和薄弱环节，推动或直接投资建设一批重大工程，确需中央政府投资的项目，建设资金纳入中央预算。
　　政府有关部门要面向社会、面向群众，广泛宣传本纲要，在全社会形成关心和参与规划实施的氛围。在制定和实施年度计划时，要贯彻落实纲要提出的目标和任务，有关部门要针对所负责领域的任务，制定具体措施。各级地方政府在经济工作中，要从实际出发，贯彻好本纲要的精神。
　　加强规划实施的跟踪分析，特别要加强对经济增长率、失业率、通货膨胀率、国际收支等宏观调控目标的监测预警，自觉接受人民代表大会及其常务委员会的监督检查。当国内外环境发生重大变化或因其他重要原因使实际经济运行严重偏离本纲要提出的目标时，国务院要提出调整方案，报请全国人民代表大会常务委员会审议批准实施。
　　继续按照“一国两制”方针和基本法，全力支持香港、澳门特别行政区政府依法施政，维护香港、澳门的长期繁荣稳定。巩固和加强香港国际金融、贸易、航运中心的地位，加强港澳台与内地的经济合作及交流。
　　“十五”计划是进入新世纪的第一个五年计划，是开始实施现代化建设第三步战略部署的第一个五年计划，也是社会主义市场经济体制初步建立后的第一个五年计划。全国各族人民要在中国共产党的领导下，紧密团结在以江泽民同志为核心的党中央周围，高举邓小平理论伟大旗帜，振奋精神，开拓进取，扎实工作，为实现“十五”计划的目标，为建设富强民主文明的社会主义现代化国家和早日完成祖国统一大业而努力奋斗！
　　附件四：

　关于《[国民经济和社会发展第十个五年计划纲要（草案）](https://www.pkulaw.com/chl/f439200c548488c1bdfb.html)》修改情况的报告

九届全国人大四次会议主席团：
　　根据人大代表和政协委员对“十五”计划《纲要》（草案）的修改意见，我们对《纲要》进行了修改完善，现将修改情况报告如下，请予审定。
　　1、将第7页第一节中“保护和提高粮食生产能力”和“稳步提高粮食生产能力”均修改为“稳定粮食生产能力”。
　　2、将第9页中“加强农村能源综合建设，发展沼气、节能灶”修改为“发展沼气、节能灶等新能源和新型节能技术，加强农村能源综合建设”。
　　3、将第9页中“精简乡镇机构和人员，切实减轻农民负担”修改为“精简乡镇机构和人员，减少村组干部补贴人数，切实减轻农民负担”。
　　4、将第10页中“增加对贫困地区的财政转移支付”修改为“加大对贫困地区的财政转移支付力度”。
　　5、将第16页中“兴建黄河沙坡头、嫩江尼尔基、淮河临淮岗、岷江紫坪铺、澧水皂市、右江百色等工程”修改为“兴建黄河沙坡头、嫩江尼尔基、淮河临淮岗、岷江紫坪铺、澧水皂市、右江百色、塔里木河及黑河整治等水利工程”。
　　6、将第18页中“开工建设龙滩、小湾、水布垭、构皮滩、三板溪、公伯峡等大型水电站，抓紧长江上游水电开发前期工作”修改为“开工建设龙滩、小湾、水布垭、构皮滩、三板溪、公伯峡、瀑布沟等大型水电站，抓紧长江上游溪洛渡或向家坝水电站开发的前期论证工作”。
　　7、将第19页中“因地制宜实施坡耕地退耕还林还草，恢复和扩大林草植被，推进防沙治沙”修改为“因地制宜实施坡耕地退耕还林还草，推进防沙治沙和草原保护，注意发挥生态的自我修复能力”。
　　8、在第20页中“促进西陇海兰新线经济带、长江上游经济带和南（宁）贵（阳）昆（明）经济区的形成”之后增加了“提高城镇化水平”。
　　9、将第20页中“进一步帮助少数民族地区加快发展”修改为“加大支持力度，加快少数民族和民族地区经济与社会全面发展”，并增加了“注意支持人口较少的少数民族的发展”。
　　10、将第23页中“为产业结构调整提供技术支撑”修改为“为产业结构调整特别是传统产业升级提供技术支撑”。
　　11、在第26页发展各级各类教育一节中增加了“重视发展儿童早期教育”。
　　12、将第26页中“加强教师队伍建设，提高师资水平”修改为“加强教师队伍建设，提高教师思想和业务素质”。
　　13、将第26页中“加大国家对教育的投入。加强县级人民政府对基础教育的统筹，加大省级人民政府对义务教育的支持力度”修改为“增加国家对教育的投入，加大中央和省级人民政府对义务教育的支持力度，加强县级人民政府对基础教育的统筹”。
　　14、将第27页中“特别是具有国际水平的学术带头人”修改为“特别是具有世界前沿水平的学科带头人”。
　　15、在第29页“加强规划与管理，搞好江河全流域水资源的合理配置，协调生活、生产和生态用水”之后增加了“加强江河源头的水源保护”。
　　16、将第29页中“建立合理的水价形成机制”修改为“建立合理的水资源管理体制和水价形成机制”。
　　17、将第29页中“加大海洋资源调查、开发、保护和管理力度，发展海洋产业”修改为“加大海洋资源调查、开发、保护和管理力度，加强海洋利用技术的研究开发，发展海洋产业”。
　　18、在第31页“抓好重点行业的污染防治，控制和治理工业污染源”之后增加了“依法关闭污染严重、危害人民健康的企业”。
　　19、将第31页中“防治不合理使用化肥、农药、农膜和超标污灌带来的化学污染”修改为“防治不合理使用化肥、农药、农膜和超标污灌带来的化学污染及其他面源污染”。
　　20、将第31页中“修改不合理的污染物排放标准，加强环境保护执法和监督”修改为“修改不合理的污染物排放标准，健全环境监测体系，加强环境保护执法和监督”。
　　21、在第32页深化国有企业改革一节中增加了“积极疏通和逐步规范企业特别是上市亏损公司退出市场的通道”。
　　22、将第33页中“凡是对外资开放的领域，原则上都允许内资进入”修改为“凡是对外资开放的领域，内资均可进入”。
　　23、在第33页“破除地方封锁”之后增加了“反对地方保护主义”。
　　24、在第36页推进行政管理体制和政府机构改革一节中增加了“发挥商会、行业协会等中介组织的作用”。
　　25、将第37页中“建立健全符合国际规范和我国国情的对外经济贸易体制”修改为“建立健全符合国际通行规则和我国国情的对外经济贸易体制”。
　　26、将第40页中“发展具有市场前景的劳动密集型产业”修改为“发展具有比较优势的劳动密集型产业”。
　　27、将第40页中“建立阶段性就业制度，发展非全日制就业、季节性就业等弹性就业形式，提倡自主就业”修改为“采取非全日制就业、季节性就业等灵活多样的就业形式，提倡自主就业”。
　　28、将第42页中“建立健全收入分配的激励机制和约束机制。对国有企业领导人和科技骨干实行年薪制和期权试点。在收入分配中要充分体现科学技术工作和经营管理的劳动价值”修改为“建立健全收入分配的激励机制，要提高国有企业高层管理人员、技术人员的工资报酬，充分体现他们的劳动价值，可以试行年薪制。对国有上市公司负责人和技术骨干还可以试行期权制。同时要建立严格的约束、监督和制裁制度”。
　　29、将第44页中“特别是加强青少年的思想政治、道德品质和法制教育”修改为“特别是加强青少年的思想政治、道德品质、心理健康和法制教育”。
　　30、在第45页第二十章加强思想道德建设中增加了“加强烈士陵园等爱国主义教育基地的保护和建设”。
　　31、将第45页中“加强图书馆、博物馆、文化馆、科技馆和青少年活动场所等文化设施建设”修改为“加强图书馆、博物馆、文化馆、科技馆、档案馆和青少年活动场所等文化设施建设”。
　　32、将第46页中“主动听取政协的意见”修改为“主动听取政协和各社会团体的意见”。
　　33、将第47页中“依法办案，反对地方保护，严格执法，公正司法”修改为“依法办案，反对地方保护主义，严格执法，公正司法”。
　　34、将第47页中“依法打击民族分裂、宗教极端势力、暴力恐怖活动、邪教和利用宗教进行的非法活动等”修改为“依法打击民族分裂活动、宗教极端势力、暴力恐怖活动、邪教和利用宗教进行非法活动”。
　　35、将第48页中“加强国防动员体系建设，增强平战转换能力”修改为“加强国防动员体系建设，增强国防后备力量，提高平战转换能力”。
　　36、在第50页整顿市场秩序中增加了“完善市场监督机制，运用现代科技手段，加大监管力度”。
　　此外，还对几处文字和标点符号做了修改。
　　有些意见没有采纳，主要考虑：一是一些必要的地方性项目，可在有关重点专项规划中反映；二是一些过于具体的问题，可在今后工作中注意解决，不宜写入《纲要》。

朱镕基
2001年3月10日

© 北大法宝：（[www.pkulaw.com](https://www.pkulaw.com/)) 专业提供法律信息、法学知识和法律软件领域各类解决方案。北大法宝为您提供丰富的参考资料，正式引用法规条文时请与标准文本核对。
欢迎查看所有[产品和服务](https://www.pkulaw.com/helps/2.html)。法宝快讯：[如何快速找到您需要的检索结果？](https://www.pkulaw.com/helps/70.html)[法宝](https://www.pkulaw.com/helps/69.html)V6有何新特色？

扫描二维码阅读原文

**原文链接：**<https://www.pkulaw.com/chl/cdab5d596bd8826dbdfb.html>
